# Effect of rhGH treatment on lipidome and brown fat activity in prepuberal small for gestational age children: a pilot study

**DOI:** 10.1038/s41598-025-89546-4

**Published:** 2025-02-08

**Authors:** Lorena González, Carolina Gonzalez-Riano, Pablo Fernández-García, Rubén Cereijo, Aina Valls, Andrea Soria-Gondek, Nativitat Real, Belén Requena, Joan Bel-Comos, Patricia Corrales, David Jiménez-Pavón, Coral Barbas, Francesc Villarroya, David Sánchez-Infantes, Marta Murillo

**Affiliations:** 1 Fundació Institut Germans Trias i Pujol, Barcelona, E-08916 Spain; 2 Centro de Metabolómica y Bioanálisis (CEMBIO), Facultad de Farmacia, Universidad San Pablo-CEU, CEU Universities, Urbanización Montepríncipe, Boadilla del Monte, 28660 Spain; 3 Department of Basic Health Sciences, University Rey Juan Carlos (URJC), Campus Alcorcón, Madrid, E-28922 Spain; 4 Departament of Biochemistry and Molecular Biomedicine, and Institut de Biomedicina (IBUB), University of Barcelona, Barcelona, Spain; 5 Centro de Investigación Biomédica en Red de Fisiopatología de la Obesidad y Nutrición (CIBEROBN), Instituto de Salud Carlos III, Madrid, E-28029 Spain; 6 Pediatric Department, Hospital Universitari Germans Trias i Pujol, Badalona, E-08916 Spain; 7 MOVE-IT Research Group, Department of Physical Education, Faculty of Education Sciences, University of Cadiz, Cadiz, Spain; 8 Biomedical Research and Innovation Institute of Cádiz (INiBICA), Cadiz, Spain; 9 Centro de Investigación Biomédica en Red of Frailty and Healthy Aging (CIBERFES), Instituto de Salud Carlos III, Madrid, E-28029 Spain

**Keywords:** Recombinant human growth hormone, Lipidome, Brown adipose tissue, Small for gestational age, Hormones, Lipidomics, Endocrinology

## Abstract

Recombinant human growth hormone (rhGH) therapy is the primary treatment for children born small for gestational age (SGA) who fail to show spontaneous catch-up growth by two or four years. While its effects on white adipose tissue are well-documented, this pilot study aimed to investigate its impact on the lipidome and the thermogenic and endocrine activities of brown adipose tissue (BAT) in SGA children following rhGH treatment. The study involved 11 SGA children divided into two groups: (a) SGA children who were not treated with rhGH (*n* = 4) and (b) SGA children who received rhGH treatment with Saizen^®^ (*n* = 7). This second group of seven SGA children was followed for 12 months after initiating rhGH treatment. Interventions included 12-hour fasting blood extraction and infrared thermography at baseline and 3 and 12 months post-treatment. Five appropriate-for-gestational-age (AGA) children served as controls. Exclusion criteria included endocrinological, genetic, or chronic diseases. Untargeted lipidomics analysis was performed using liquid chromatography-mass spectrometry (LC-MS), and serum biomarker levels were measured using ELISA assays. Serum lipidomic analysis revealed that free fatty acids (FFAs) increased to levels close to those of the AGA group after three months of rhGH administration, including polyunsaturated fatty acids, correlating with reduced leptin levels. Elevated levels of 1a,1b-dihomo-PGJ2 and adrenic acid suggested potential aging markers. rhGH treatment also significantly reduced meteorin-like (METRNL) and monocyte chemoattractant protein-1 (MCP1) serum levels to control levels. rhGH influences the serum lipidome, promoting changes in maturation and metabolism. Further research is required to clarify the direct effects of rhGH on specific lipid species and batokines, potentially addressing metabolic disturbances linked to obesity and aging.

**Supplementary Information:**

The online version contains supplementary material available at 10.1038/s41598-025-89546-4.

## Introduction

Children with birth weight and/or length below two standard deviations (SD) for the gestational age and sex of the population are categorized as small for gestational age (SGA) and represent 3.1–5.5% of the population^1^. While most SGA infants typically experience spontaneous catch-up growth during the early stages, about 10% of these children do not and continue exhibiting short stature throughout their childhood and adulthood^2^. Recombinant human growth hormone (rhGH) treatment was approved in Europe in 2003 as a treatment for short children born SGA if they meet internationally established criteria: no catch-up growth at four years of life and height < − 2.5 SD^3^. The body composition of SGA infants is different from that of appropriate-for-gestational-age (AGA) children, with decreased total body fat, lean mass, and bone mineral content^4^.

rhGH is a hyperglycemic hormone, and its ability to induce white adipose tissue (WAT) lipolysis has been documented for over eight decades^5^. Notably, rhGH treatment in SGA children reduces subcutaneous WAT without affecting abdominal WAT, increases muscle mass, and normalizes bone mineral density. At the molecular level, rhGH influences key adipokines, decreasing leptin and high-molecular-weight adiponectin^6^while concurrently improving lipid parameters by reducing the abnormally elevated serum levels of total cholesterol (TC), non-high-density lipoprotein (HDL) cholesterol, and low-density lipoprotein (LDL) cholesterol in SGA children^7–10^. Despite these established effects, the impact of rhGH treatment on children’s lipid profiles has not been studied in detail to date.

Brown adipose tissue (BAT) is the main thermogenic organ in mammals, being much more prevalent in children than in adults^11^. BAT plays a pivotal role in modulating systemic metabolism due to its capacity to oxidize metabolic substrates to produce heat, but also through the secretion of batokines, influencing glucose homeostasis in an endocrine manner^12^. BAT receptors include, among others, those for insulin, glucose, fatty acids, estrogen, prolactin, growth hormone, and insulin-like growth factor-1 (IGF-1)^13^.

The effects of rhGH on WAT are well established; nevertheless, very little is known about its effect on BAT. Some studies presented data highlighting the function of brown fat in adolescents^14^and altered BAT activity in adults born SGA^15,16^. However, to our knowledge, no studies have investigated the influence of rhGH on BAT in prepubertal SGA children. This study aims to fill this gap by comprehensively evaluating the effects of rhGH treatment on the lipid profile and BAT activity in SGA children.

## Materials and methods

### Study population and study design

The study population consisted of 11 SGA children attending the Pediatric Endocrinology Unit at the Germans Trias i Pujol Hospital, Badalona, Spain, between April 2021 and November 2022.

Inclusion criteria for participating in this study were:

- children aged between 3 and 9 years who were born SGA, defined as birth weight or length under 2 SD for gestational age and sex using Spanish growth standards.
- children with prepubertal status defined as Tanner stage I breast development for girls and testicular volume less than 4 mL for boys.

Exclusion criteria were any other endocrinological disease, genetic syndrome, or major congenital or chronic disease.

Participants classified as SGA (*n* = 11) were divided into two groups: those without rhGH administration (*n* = 4) and those receiving rhGH (SGA-GH; *n* = 7), who were followed for 3 and 12 months after initiating treatment. Additionally, five AGA children served as the control group. According to local standards, rhGH treatment was indicated if they had a height < −2.5 SD at the time of evaluation. All patients requiring rhGH received daily treatment with rhGH (Saizen^®^) using the EasyPodSystem device within the usual range of doses (0.23–0.30 mg/kg/week sc).

## Data acquisition

**Fig. 1:**
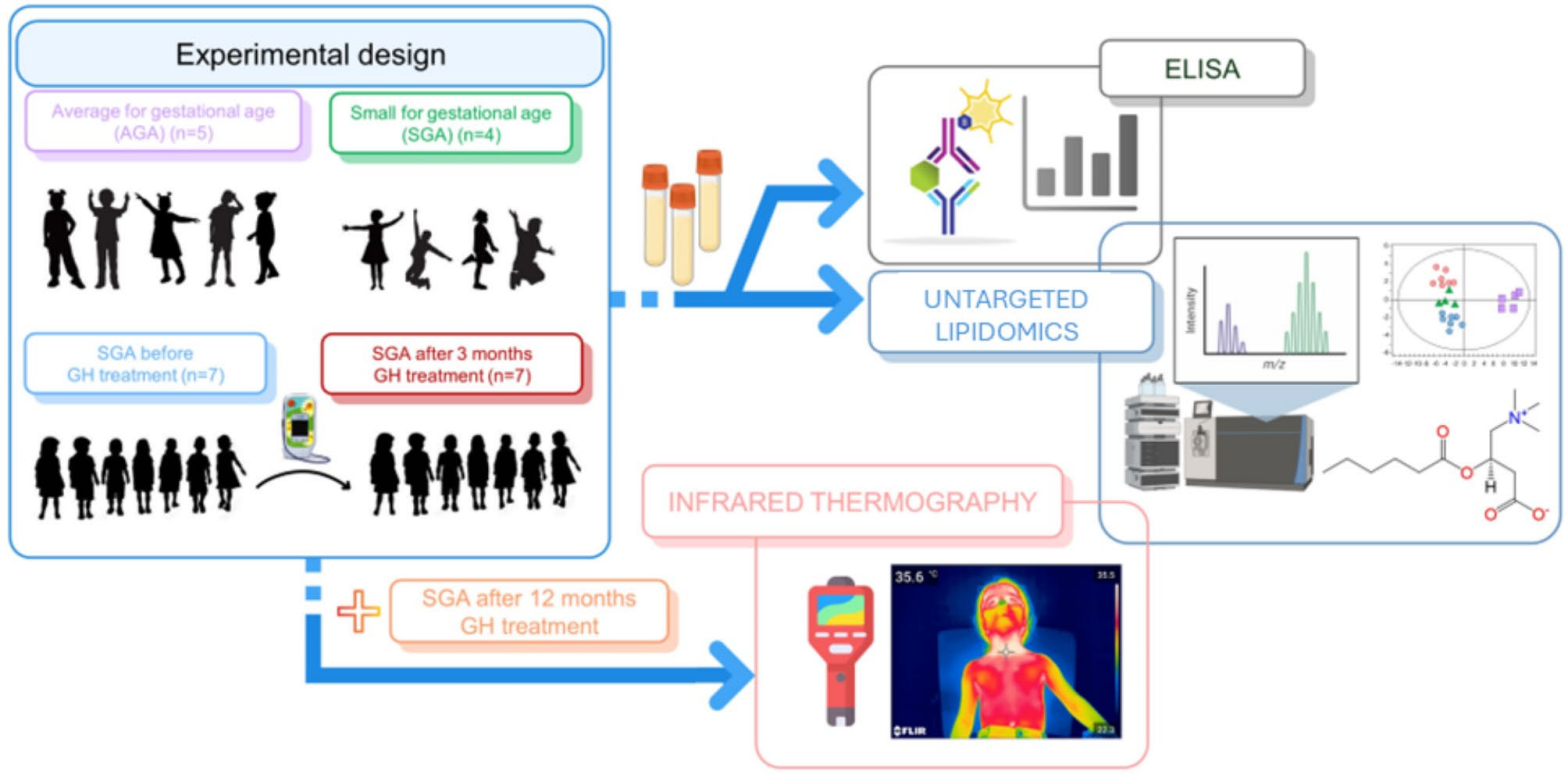
Study design. Schematic overview of the participant groups compared and the various approaches of this project, including analytical techniques such as untargeted lipidomics and serum biomarker analysis performed.

All SGA participants were attended at baseline, and if receiving rhGH treatment, they were attended 3 and 12 months after initiating treatment. At each visit, height (Harpenden stadiometer), weight, body mass index (BMI), and adherence data were collected using the EasypodConnect^®^ app. The response to rhGH treatment was mainly assessed according to the growth velocity calculated between the different visits. A 12-h fasting blood extraction and infrared thermography analysis were performed at baseline in all patients, and at 3 and 12 months in the case of rhGH treatment (Fig. 1) to obtain biochemical parameters, including batokine levels and BAT activity.

A third group of five AGA children of normal stature and similar age, not receiving rhGH, served as the control group.

The Institutional Ethics Committee (Germans Trias i Pujol CEIC) approved the study. It was made in accordance with the Declaration of Helsinki. All participants gave their written informed consent before collecting clinical data and samples.

*Lipidomic analysis*.

1. *Sample treatment.*

A single-phase extraction method was performed for lipid extraction, as previously described^17^. Briefly, 50 µL of serum sample was mixed with 175 µL of methyl-tert-butyl-ether (MTBE) and 175 µL of cold methanol (−20 ºC), which contained 2.30 ppm of sphinganine (d17:0) as the internal standard (IS) for electrospray ionization (ESI) (+), and 4.60 ppm of palmitic acid-d31 as the IS for ESI (–). The mixture was vortexed thoroughly for 1 h at room temperature and then centrifuged (16,100 x *g*, 5 min, 15 ºC) before being transferred into sample vials with glass inserts for Ultra-HPLC-mass Spectrometry (UHPLC-MS) analysis. To ensure the robustness and reproducibility of the analysis, quality control samples (QCs) were prepared by pooling equal volumes of each serum sample. These QCs underwent the same processing steps as the other study samples. The samples were subsequently randomized, with QCs injected at the start, after every five experimental samples, and at the end of the batch. Additionally, four blank samples were prepared alongside the other samples, following an identical lipid extraction procedure. These blank samples were then analyzed at the beginning and end of the analytical sequence to detect any common contaminations. Finally, serum extracts were measured using an Agilent 1290 Infinity II UHPLC system connected to an Agilent 6545 quadrupole time-of-flight (QTOF) mass spectrometer (Agilent Technologies, Santa Clara, CA, USA), both in positive and negative ion modes, employing a previously established analytical condition^18^. Further details, including standards, chemicals, reagents, solvents, and analytical conditions, are provided in the Supporting Information.

## Lipidomic data analysis

The data obtained from LC-MS analysis underwent a data-cleaning process to remove background interference and irrelevant ions. This cleaning was performed via a recursive analysis using MassHunter Profinder software (version B.10.0.2, Agilent Technologies, Santa Clara, CA, USA). Initially, the Molecular Feature Extraction (MFE) algorithm performed chromatographic deconvolution to build all the mass spectral data features, which are the sum of coeluting ions that are related by charge-state envelope, isotopologue pattern, and/or the presence of different adducts and dimers in the analyzed samples. Simultaneously, the MFE aligned these molecular features across all study samples using their mass and retention time (RT) to create a unified spectrum for each compound group. Subsequently, the MFE results were employed for Recursive Feature Extraction. The Batch Find by Ion extraction (FbI) algorithm utilized the median mass, median RT, and a composite spectrum derived from the aligned features to enhance reliability^18^. To identify coeluting adducts of the same feature, the following adducts were selected: [M + H]^+^, [M + Na]^+^, [M + K]^+^, [M + NH_4_]^+^, and [M + C_2_H_6_N_2_ + H]^+^ in LC-ESI(+)-MS; [M-H]^-^, [M + Cl]^-^, [M + CH_3_COOH-H]^-^, and [M + CH_3_COONa-H]^−^ in LC-ESI(-)-MS. Additionally, the neutral loss (NL) of water was considered for both ion modes.

## Data normalization and statistical analysis

Before conducting statistical analysis, data normalization and filtering were implemented. Features that exhibited mean blank values exceeding 10% of the mean values in the samples were discarded^19^. Initially, the coefficient of variation (CV) for both ISs was computed. Subsequently, the raw data matrices were normalized based on the intensity of their respective IS to correct for undesired variability associated with sample preparation and analytical run. Their CV then guided the selection of features in the QCs, and a cutoff threshold of 20% was established for the CV values of lipids found in the QC samples in the LC-MS dataset.

Differences among groups were examined through both univariate (UVDA) and multivariate (MVDA) data analyses. In the context of UVDA, differences among the four study groups were assessed for each lipid using Matlab (R2018a, MathWorks) by applying pairwise analyses using the Mann-Whitney U test (*p* ≤ 0.05)—after normality testing using the Shapiro-Wilk test—to determine the significance of lipids in various comparisons (SGA vs. AGA; SGA-GH 0 m vs. AGA; SGA-GH 3 m vs. SGA; SGA vs. SGA-GH 0 m, SGA vs. SGA-GH 3 m). The Wilcoxon signed-rank test was used for the SGA-GH 0 m vs. SGA-GH 3 m comparison. Finally, the Benjamini–Hochberg correction test was used to inspect the false discovery rate at an α = 0.05 level. Compounds with Mann-Whitney U test *p*-values slightly above 0.05 were retained, enhancing the biological insights of the study based on their significant *p*-value in at least one of the comparisons and considering that their levels followed the same trend as the lipid species within their class. For MVDA (SIMCA *P* + 16.0), Pareto scaling and logarithmic transformation were applied before generating unsupervised principal component analysis (PCA-X), partial least square-discriminant analysis (PLS-DA), and orthogonal partial least square-discriminant analysis (OPLS-DA) models. The groups were then compared using the OPLS-DA model to maximize class differentiation and explore the influential factors among the variables. Variable influences on projection (VIP) values were calculated using the OPLS-DA models, retaining lipids with a VIP value of ≥ 1 and a jackknife confidence interval value other than zero. Finally, the OPLS-DA models were validated through cross-validation and the CV-ANOVA tool provided by SIMPA-P + software. PCA, PLS-DA, and OPLS-DA models and their corresponding quality parameters are described in Supplementary Figure S1.

## Lipid annotation

Accurately annotating the lipid species and other compounds detected by LC-MS is vital for providing a comprehensive biological interpretation of the results. The annotation process^18^ consisted of three key steps: (i) initial tentative identification of lipid features based on MS1 data using our online tool CEU Mass Mediator (CMM) (http://ceumass.eps.uspceu.es/mediator/)^20^, (ii) reprocessing of the raw LC-MS/MS data with Lipid Annotator software (Agilent Technologies Inc., Santa Clara, CA, USA)^21^, and finally, (iii) manual MS/MS spectral interpretation using Agilent MassHunter Qualitative software (version 10.0), comparing RT and MS/MS fragmentation to the spectral data available in several databases^22–24^. Detailed information about the lipid annotation process can be found in the Supporting Information. The lipid nomenclature convention used herein for the lipid species reported follows the latest update of the shorthand annotation^25^. Heatmaps illustrating lipid changes were generated using MetaboAnalyst 6.0 (https://www.metaboanalyst.ca/). A heatmap was generated for each lipid category (glycerophospholipids, fatty acyls, sphingolipids, and glycerolipids) to display the differential expression of lipid species across study groups and time points.

### Infrared thermography

BAT thermogenic activation capacity elicited by cold stimulus was assessed using infrared thermography (IRT) with a cold stimulation protocol, as previously described by Irene Piquer et al.^26^. All participants underwent IRT with a cold stimulation protocol at baseline and, if receiving rhGH treatment, after 3 and 12 months of treatment.

Briefly, for IRT acquisition, participants were placed in a specific room where the body area object under evaluation was uncovered. The patient sat for 5 min in a thermoneutral ambient for acclimatization (24.3 ± 1.6 °C).

For the cold protocol, three thermal images were taken of each patient using a FLIR T420 infrared camera (FLIR T420 Systems AB, Sweden), with a thermal sensitivity of 0.05 °C and resolution set at 320 × 240 pixels. The first image was taken with an aluminum foil phantom (1 m away) to obtain a measurement of the reflected temperature for each set of images; at this moment, ambient temperature and relative humidity were also registered for each set. For the second thermal image, the participants remained seated in an upright position, with their arms relaxed on both sides of their legs. The camera was placed 1 m from the midpoint of the chair for these images. For the third image, patients were asked to put their left hand in cold water (17 °C) for 5 min to stimulate BAT activation, after which the thermographic picture was taken. The temperature was controlled with a thermometer, and cold blocks were used to maintain the 17 °C when necessary.

Thermal data were extracted from IRT pictures using a region of interest (ROI)-based approach. The ROIs were manually drawn in the images on the supraclavicular and sternum region using the FLIR ResearchIR Max software version 4.40.6.24 for Windows (FLIR Systems Inc., North Billerica, MA, USA). All analyses were adjusted for atmospheric temperature, distance from the participant, and relative humidity, which, as previously stated, were recorded at the beginning of each IRT session. Moreover, the reflected temperature was obtained by placing a rounded ROI on the aluminum foil phantom of the first thermal image and retaining the mean value (°C) for adjustments. Emissivity was set at 0.98 (human skin) for all thermographic images. Each ROI’s minimum, maximum, and mean values were retained as variables. The temperature in the supraclavicular region was normalized to the sternum region temperature for each participant at all time points before and after surgery.

We calculated the delta Supraclavicular T-Sternum T in thermal image numbers 2 (0 min) and 3 (5 min after cold stimulation), obtaining a “ΔT0” value for thermal image 1 and a “ΔT5” value for thermal image 2^26^.

Next, we calculated the delta “ΔT0-5min,” representing the value of BAT thermogenic activation capacity elicited by cold stimulus^26^.

We repeated this same sequence 3 and 12 months after rhGH treatment and compared the values of BAT thermogenic activation capacity by cold stimulus with the results obtained before treatment.

## Serum samples

All samples were stored at − 80 ºC. Glucose, insulin, HbA1c, total cholesterol, LDL cholesterol, HDL cholesterol, triglycerides (TG), thyroid-stimulating hormone (TSH), free thyroxine-4 (T4), alanine transaminase (ALT), aspartate transaminase (AST), IGF1, insulin-like growth-factor binding protein-3 (IGFBP3), and sex hormone-binding globulin (SHBG) were determined in the clinical laboratory of the Germans Trias i Pujol Hospital (Badalona, Spain).

Circulating levels of resistin, tumor necrosis factor-alpha (TNF-α), monocyte chemoattractant protein-1 (MCP-1/CCL-2), Leptin, interleukin-8 (IL-8), and interleukin-6 (IL-6) were determined using a soluble protein quantification system (Milliplex MAP Human Adipokine Magnetic Bead Panel 2 HADK2MAG-61 K-05 and Milliplex MAP Human Adipocyte Magnetic Bead Panel HADCYMAG-61 K-05 Millipore, Billerica, MA, USA). Human-specific ELISA assays were employed for quantification of Chemokine (C-X-C motif) ligand 14 (CXCL14) (ELH-CXCL14-1, Raybiotech), Fibroblast growth factor-21 (FGF21) (RD191108200R, Biovendor), Bone morphogenetic protein-8b (BMP8B) (MBS944757, MyBioSource), Growth-and-differentiation factor-15 (GDF15) (Duo set ELISA DY957 Bio-Techne and Duo set ELISA ancillary reagent kit 2 DY008 Bio-Techne), Adiponectin (EZHADP-61 K, Merck Life Science) and Meteorin-like protein (METRNL) (Human Meteorin-like/Metrnl Duo Set ref DY7867-05 Bio-techne; Duo Set Elisa Ancillary Reagent Kit 2 ref DY008B Biotechne) were determined at the Department of Biochemistry and Molecular Biomedicine, and Institut de Biomedicina (IBUB), University of Barcelona (Spain).

### Statistical analysis

Results are expressed as mean ± SD. We assumed the normality of datasets, any outliers were evaluated with the ROUT test, and Pearson’s r was used to discard any correlation with sex or age among the different study groups. A two-tailed unpaired Student’s t-test was used for normal two-variable comparisons among the different study groups, and Welch’s correction was routinely implemented in case of significant variance divergences. One-way ANOVA followed by Tukey’s *post-hoc* test was used for parametric comparisons between the three times points of the cohort receiving rhGH treatment. Statistical analyses were implemented in GraphPad Prism 8 (GraphPad Software Inc., USA). The significance threshold was set as *p* < 0.05 in all cases.

## Results

### Clinical parameter modulation after rhGH treatment

A cohort of 11 SGA children (five boys and six girls) who met the established inclusion criteria entered the clinical study; seven initiated rhGH treatment and were prospectively followed up for 12 months. Clinical and anthropometric data of the study participants are summarized in Supplementary Table S1−2. The response to rhGH treatment was modest, with an improvement in mean growth velocity of almost 2 cm/year and a mean height gain of 0.45 SD at one year of treatment. The adherence to rhGH treatment was correct (mean = 93.8%) (Supplementary Table S3).

*Lipid species are altered after rhGH treatment.*

**Fig. 2A:**
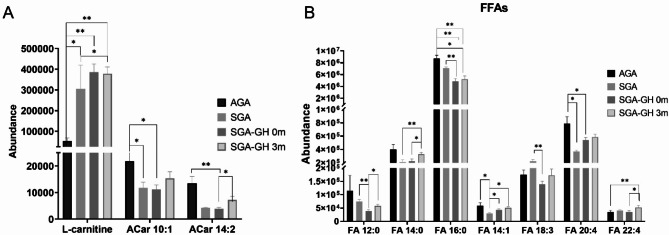
B. The bar plots illustrate the changes in lipid levels between the different groups. Each bar represents the mean lipid level for (**A**) L-carnitine and acylcarnitines, and (**B**) free fatty acids (FFAs) within a specific group, with error bars indicating the Standard Error of the Mean (SEM) (AGA, *n* = 5; SGA *n* = 4, SGA-GH 0 m, *n* = 7; SGA-GH 3 m, *n* = 7). Statistical significance was determined using the Mann-Whitney U test, *p* < 0.05. *p ≤ 0.05; **p ≤ 0.01.

**Fig. 3A-B:**
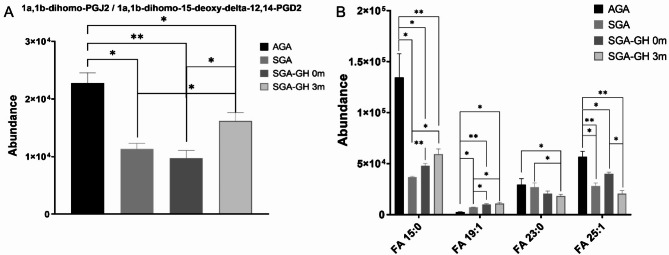
**The bar plots depict the changes in (A) 1a**,**1b-dihomo-PGJ2 / 1a**,**1b-dihomo-15-deoxy-delta-12**,**14-PGD2 and (B) odd-chain fatty acid levels at different growth stages and rhGH treatment**. Each bar represents the mean lipid level for a specific group, and error bars denote the standard error of the mean (SEM). Statistical significance was determined using the Mann-Whitney U test, *p* < 0.05. *p ≤ 0.05; **p ≤ 0.01.

**Fig. 4:**
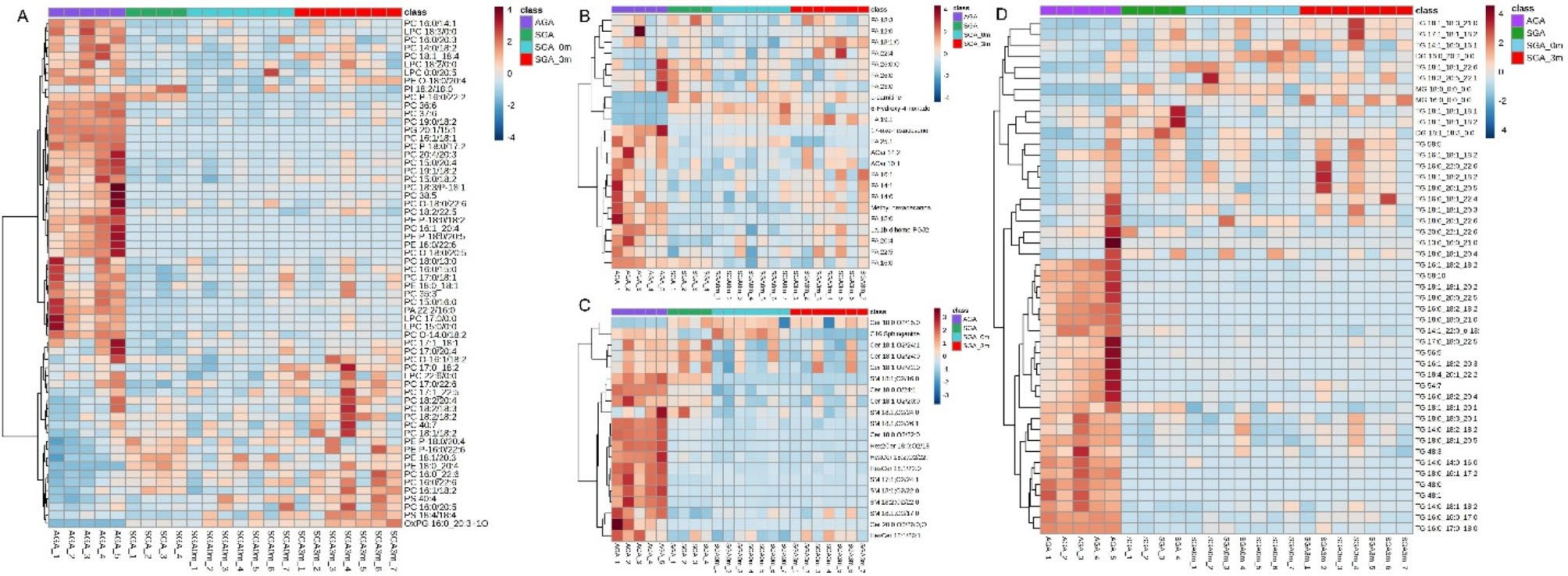
**Heatmaps of Lipid Changes by Lipid Category**. Heatmaps illustrating the differential expression of lipid species across study groups and time points. Lipid species detected in the study were categorized into distinct categories: (**A**) glycerophospholipids, (**B**) fatty acyls, (**C**) sphingolipids, and (**D**) glycerolipids. Each heatmap represents the relative abundance of lipid species, with color gradients indicating the degree of change. Red and blue colors represent increased and decreased lipid levels, respectively. Compared with AGA controls, the heatmaps display lipid changes for SGA children before and after three months of rhGH treatment. These visualizations highlight significant lipid alterations associated with rhGH treatment and provide insights into the lipidomic profile changes influenced by the therapy.

Using the LC-MS ESI(+) and LC-MS ESI(−) approaches, 1058 and 421 features were detected, respectively. After data normalization, filtration by CVs in QCs (< 20%), and blank subtraction, 753 and 393 features were obtained, respectively. Next, based on the VIP threshold (VIP > 1.0) and *p* ≤ 0.05 in the UVDA, 164 lipid species were found to be modulated (Supplementary Table S4 and S5). PLS-DA was then employed to reveal the global lipidomic changes related to the different study groups. The PLS-DA model clearly discriminated between the four groups, displaying good quality parameters (explained variance, R2 ≥ 0.6; predicted variance, Q2 ≥ 0.4), with differences among them < 0.3 (Supplementary Figure S1). Several lipid classes, including acylcarnitines, fatty acyls, glycerophospholipids, glycerolipids, and sphingolipids, were significantly different among the groups and rhGH treatment subgroups (Figs. 2A-B and 3A-B). Finally, the generated heatmaps were used to highlight key lipid species exhibiting notable changes in response to the treatment, providing insights into the lipidomic alterations influenced by rhGH administration (Fig. 4). The lipidomics raw data are available in CEU ReI platform (access handle https://hdl.handle.net/10637/17905).

*rhGH treatment showed no effect on BAT thermogenic activity and modest changes in batokine*,* adipokine*,* and cytokine levels.*

When comparing SGA children before and three months after rhGH treatment, no statistically significant differences were found in the thermogenic activation of BAT after cold exposure. A representative IRT image of one participant at each time point of the project is shown in Supplementary Figure S2, and quantitative data from the entire participant cohort is summarized in Supplementary Table S4.

**Fig. 5A-C:**
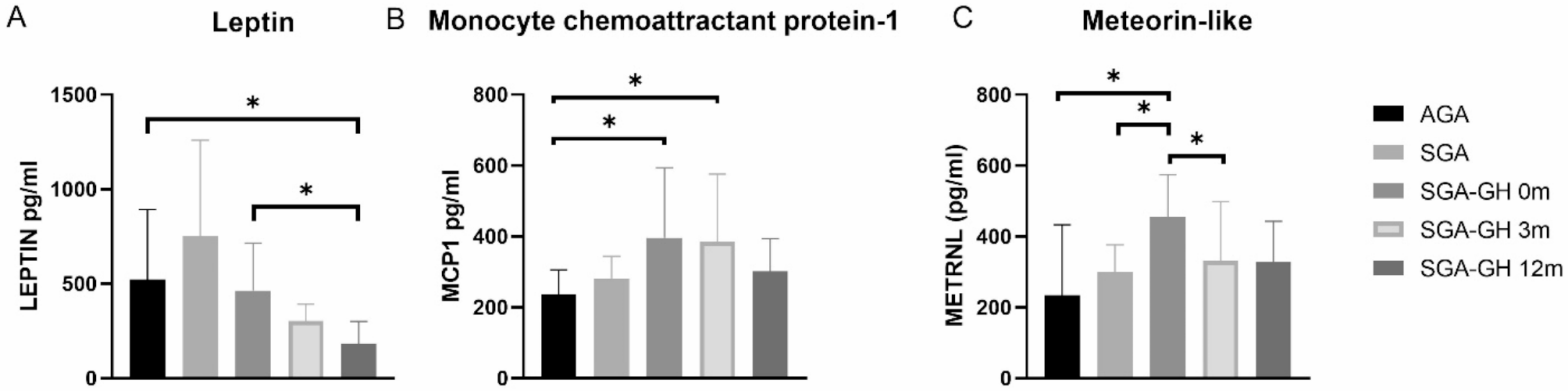
**Serum level profiles at baseline and after 3 and 12 months of rhGH treatment**. Circulating (**A**) Leptin, (**B**) Monocyte chemoattractant protein-1 (MCP1), and (**C**) meteorin-like (METRNL) levels in AGA and SGA children untreated with rhGH and rhGH-treated SGA children at baseline, 3 and 12 months after rhGH administration. Data shown are means +/- SD. A two-tailed unpaired Student’s t-test was used to compare circulating protein levels in AGA, untreated SGA, and rhGH-treated SGA groups at baseline. One-way paired ANOVA was used to compare circulating protein levels in the SGA group at baseline, 3 and 12 months after rhGH treatment. *p ≤ 0.05; **p ≤ 0.01.

Circulating serum adiponectin, BMP8b, Resistin, GDF15, CXCL14, and FGF21 levels showed a tendency to be modulated after rhGH treatment; however, no significant changes were observed (Supplementary Figure S3). Circulating serum IL-6 and IL-8 levels also showed a tendency to decrease 3 and 12 months after initiating treatment (Supplementary Figure S3). Serum leptin levels from SGA-GH children before starting rhGH treatment did not differ from the control group. However, we observed a statistically significant decrease in leptin levels of SGA-GH children after 12 months of rhGH treatment (Fig. 5A).

In addition, we also found elevated serum levels of MCP1 (Fig. 5B) and METRNL (Fig. 5C) in SGA-GH children compared with the control group. These differences disappeared after 12 months of rhGH treatment in the case of MCP1 (Fig. 5B) and after three months in the case of METRNL (Fig. 5C).

## Discussion

SGA children exhibit a remarkable degree of heterogeneity in many aspects. A significant proportion of this diverse group maintains a short stature during childhood and eventually achieves a low adult height, which is why rhGH is indicated as a treatment. To begin with, it must be admitted that the response to rhGH treatment in our cohort was modest as regards changes in height and growth velocity. This is remarkable since the treatment protocol was adhered to, and IGF1 levels increased. Notably, treatment adherence was diligently confirmed through the direct extraction of data from the administration device, ensuring accurate knowledge of the precise amount of rhGH administered and affording greater reliability to the study data.

A noteworthy effect of rhGH administration in humans is the substantial increase in free fatty acids (FFAs) within 1–2 h, indicative of stimulated lipolysis and ketogenesis^5,27–29^. Given the significant role of BAT as a regulator of the circulating lipid pool in both mice and humans^28^, we extended our investigation beyond the lipidome, and the effect of rhGH on BAT was studied. In addition to assessing BAT thermogenic activation, we examined its secretory capacity and overall pattern of adipokine and cytokine status.

**Fig. 6:**
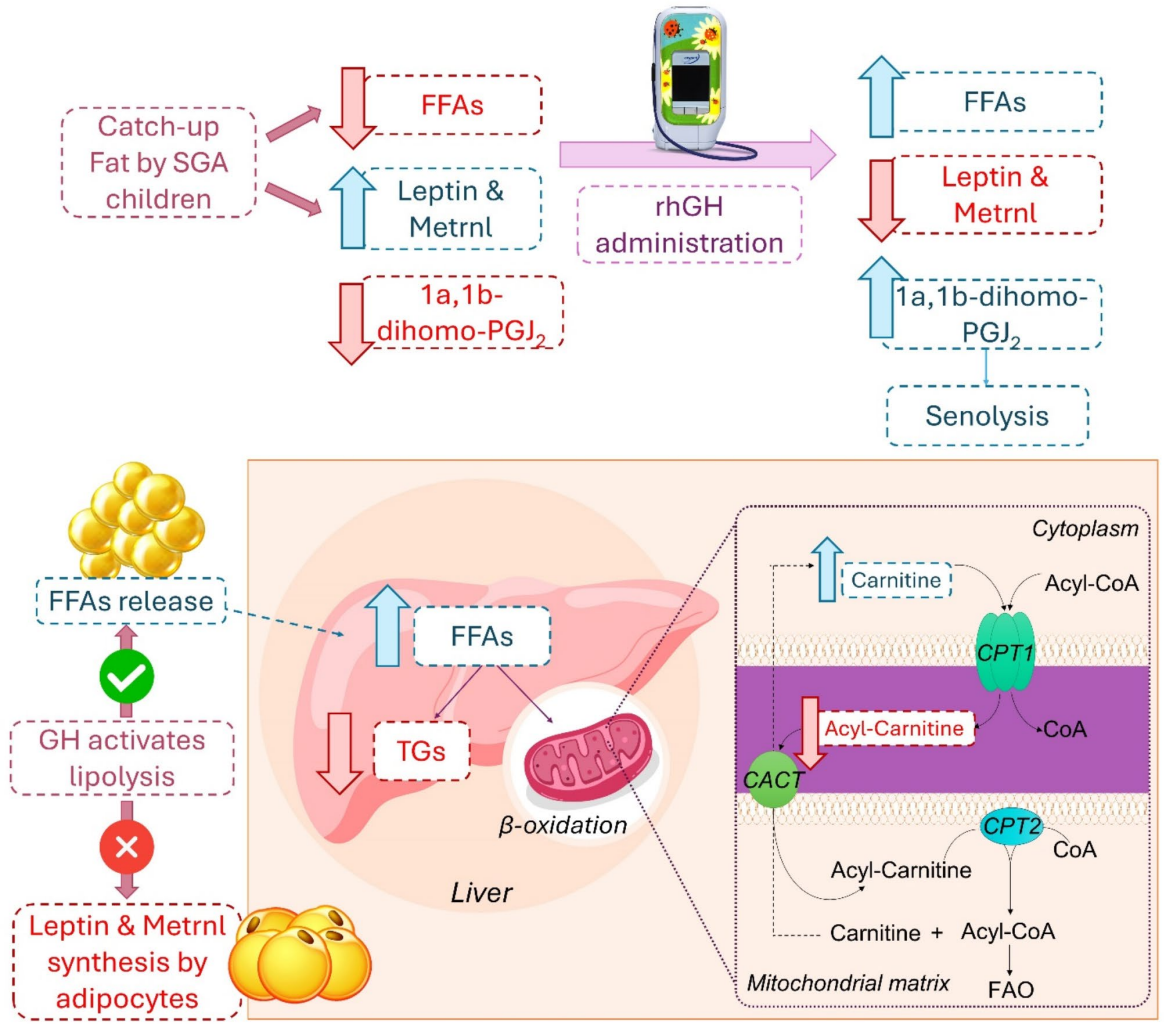
**Potential mechanisms underlying the metabolic shifts detected before and after rhGH administration compared with AGA children**. The figure highlights key alterations, including increased free fatty acid (FFA) levels approaching those seen in AGA children, suggesting enhanced lipolysis and metabolic activity. Elevated levels of 1a,1b-dihomo-PGJ2 are indicative of potential aging markers and accelerated maturation processes. Increased carnitine levels facilitate fatty acid transport into mitochondria for β-oxidation, enhancing energy production. Conversely, decreased acylcarnitines reflect improved fatty acid utilization and mitochondrial function. Additionally, lower triacylglyceride (TG) levels post-treatment indicate altered lipid storage and metabolism. Arrows indicate the direction of change (↑ increase, ↓ decrease) in these metabolic markers, underscoring the dynamic metabolic adaptations in response to rhGH therapy and providing insights into its impact on lipid homeostasis and endocrine function in SGA children.

No significant differences in BAT thermogenic activation were found by IRT; nonetheless, we did detect remarkable lipid changes when comparing SGA children at baseline and three months after rhGH treatment, including a remarkable increase in FFAs, which reached concentrations close to control levels. Additionally, we detected elevated levels of carnitine and acylcarnitines after rhGH treatment (Fig. 2A). However, compared with AGA, acylcarnitines levels were lower in SGA with or without rhGH treatment (Fig. 2A). The increase in plasma acylcarnitines derived from lipolysis in WAT has been reported to activate nuclear receptor HNF4α in liver, which is necessary for acylcarnitine production^30–32^ (Fig. 6).

Regarding TGs, we observed a general downregulation of their levels. However, 11 TG species were found to be greatly upregulated, especially after rhGH treatment (Fig. 4). We also observed a notable reduction in the levels of multiple essential FFAs in the SGA and SGA-GH groups at baseline. After three months of treatment, the levels of FFAs in the SGA-GH group tended to increase, being close to those of the control group (AGA) (Fig. 2B). Omega-3 polyunsaturated fatty acids (PUFAs)—a class of FFAs—are integral components of cell membranes and play pivotal roles in various physiological processes, including cardiovascular health, brain function, and inflammation. Research has shown that supplementation with omega-3 PUFAs induced a reduction in leptin levels^33^. According to this, leptin levels tended to be downregulated after three months of rhGH treatment in SGA, reaching significant differences 12 months after rhGH treatment. On the other hand, after three months of rhGH treatment in our study, we detected an increase in the levels of 1a,1b-dihomo-PGJ_2_ / 1a,1b-dihomo-15-deoxy-delta-^12,14^-PGD2 (Fig. 3A) and adrenic acid (FA 22:4) (Fig. 2B). It was postulated that senescent cells produce a variety of oxylipins, a class of biologically active lipids resulting from the oxygenation of PUFAs. Notably, dihomo-prostaglandins, less explored derivatives of adrenic acid, represent the principal prostaglandins synthesized by senescent cells. Among these derivatives, dihomo-15d-PGJ_2_serves as an intracellular biomarker of senescence, and its release from cells serves as a hallmark of senolysis, both in culture and in vivo^34,35^. Therefore, the accelerated bone maturation induced by rhGH treatment might be involved in the increase of these markers of aging.

Recent research findings revealed a negative correlation between the presence of circulating C15:0/C17:0 fatty acids and the risk of disease^36^. Notably, obesity has been linked to reduced serum levels of odd-chain fatty acids (OC-FAs), particularly C15:0, C17:0, and C19:0. Moreover, research has shown that higher serum levels of 15:0 are inversely related to the incidence of type 2 diabetes (T2DM), and plasma phospholipid 15:0 is inversely associated with the development of cardiovascular disease^37^. Additionally, OC-FAs, particularly FA 15:0 and FA 17:0, may have a positive impact on cardiovascular health. Higher levels of these lipid species in the bloodstream have been linked to a reduced risk of coronary heart disease^38^. In our study, we observed elevated levels of various lipid classes containing OC-FAs, notably 15:0 and 19:1, in SGA infants who underwent three-month rhGH treatment (Fig. 3B). This suggests that rhGH treatment could protect against the risk of cardiovascular disease.

As previously described^5,6^, we found that rhGH treatment decreased circulating serum leptin levels, being significantly different after 12 months of rhGH administration (Fig. 5A). Interestingly, as previously shown by Mostowik et al.., the reduction in leptin levels that we observed aligned with the increase in PUFAs detected (Fig. 3B)^33^.

Regarding the endocrine role of WAT and BAT, we found a significant reduction in circulating MCP1 levels after 12 months of rhGH administration (Fig. 5B). In contrast, in vitro rhGH treatment increased MCP1 in human mature adipocytes after 3 and 12 h, both in subcutaneous and visceral adipocytes^39^. The in vitro positive effects of rhGH administration on MCP1 synthesis have also been described in 3T3-L1 adipocytes^40^. Nevertheless, to our knowledge, the present study is the first to have assessed the in vivo global body effect of one-year rhGH injection treatment in children. Moreover, in our study, a significant decrease in METRNL was observed in SGA children after just three months of rhGH treatment. The circulating levels of this molecule reached similar concentrations to those found in AGA children (Fig. 5C), indicating a potential effect of rhGH treatment in the modulation of METRNL. The hormonal factor METRNL is induced either in skeletal muscle after exercise or in adipose tissue following cold exposure and is present in the circulation. It has been associated with protection against cardiac dysfunction^41^. Moreover, García-Beltrán et al. described that circulating METRNL levels are elevated in the first year of life and correlate with neonatal BAT activity^42^. Thus, it was proposed as a potential circulating biomarker of BAT activity in early life^42^.

### Strengths and limitations

The strength of our study lies in the novel application of a lipidomic approach, combined with batokine analysis, to investigate the impact of rhGH treatment on prepubertal SGA children. This is the first time lipidomic analysis has been assessed in this context and population. Our model is robust, and groups were clearly stratified into different cohorts. However, we acknowledge our limitations. First, our sample size may not be sufficient to draw conclusive results; hence, this is a pilot study. Secondly, the lack of data from the AGA control group one year after the baseline hampers comparisons between the SGA group after 12 months of rhGH treatment and the AGA group. Therefore, further analysis is needed to draw inferences regarding the long-term effects of rhGH treatment.

## Conclusion

In summary, the changes in the lipidomic profile and endocrine molecules, as reported in this pilot study, hint at a potential effect of rhGH treatment on the maturation process and metabolism of prepubertal children. The innovative approaches we have employed to decipher the direct effect of rhGH and the molecular mechanisms involved in these specific lipid species and batokine alterations could potentially pave the way for the identification of new targets to address metabolic disturbances associated with obesity and aging.

## Electronic supplementary material

Below is the link to the electronic supplementary material.

Supplementary Material 1

## Data Availability

Some or all datasets generated and/or analyzed during the current study are available in CEU ReI platform (access handle https://hdl.handle.net/10637/17905).

## Abbreviations

(rhGH): Recombinant human growth hormone
(SGA): small for gestational age
(BAT): brown adipose tissue
(WAT): white adipose tissue
(TC): total cholesterol
(IGF-1): insulin growth factor-1
(MTBE): methyl-tert-butyl-ether
(QCs): quality control samples
(QTOF): quadrupole time-of-flight
(MFE): Molecular Feature Extraction
(RT): retention time
(NL): neutral loss
(CV): coefficient of variation
(IS): internal standards
(UVDA): univariate data analysis
(MVDA): multivariate data analyses
(PCA-X): unsupervised principal component analysis
(PLS-DA): partial least square-discriminant analysis
(OPLS-DA): orthogonal partial least square-discriminant analysis
(VIP): Variable influences on projection
(CMM): CEU Mass Mediator
(IRT): infrared thermography
(FFAs): free fatty acids
(TNF-α): tumor necrosis factor-alpha
(MCP-1/CCL-2): monocyte chemoattractant protein-1
(IL-8): interleukin-8
(IL-6): interleukin-6
(CXCL14): Chemokine ligand 14
(FGF21): Fibroblast growth factor-21
(BMP8B): Bone morphogenetic protein-8b
(GDF15 ): Growth-and-differentiation factor-15
(METRNL): Meteorin-like protein
(OC-FAs): odd-chain fatty acids
(T2DM): type 2 diabetes
