# Supplementary material for: Effect of rhGH treatment on lipidome and brown fat activity in prepuberal small for gestational age children: a pilot study
Source: Sci Rep. 2025 Feb 8;15:4710. doi: 10.1038/s41598-025-89546-4 (PMC11807155; doi:10.1038/s41598-025-89546-4)
Supplement: Supplementary file 1 — Supplementary Material 1 [file 41598_2025_89546_MOESM1_ESM.pdf]

Effect of rhGH Treatment on Lipidome and Brown Fat Activity in Prepuberal Small-for-Gestational-Age Children: A Pilot Study

Lorena González[1*], Carolina Gonzalez-Riano[2*], Pablo Fernández-García[3], Rubén Cereijo[4,5], Aina Valls[6], Andrea Soria-Gondek[6], Nativitat Real[6], Belén Requena[2], Joan Bel-Comos[6], Patricia Corrales[3], David Jiménez-Pavón[7,8,9], Coral Barbas[2], Francesc Villarroya[4,5], David Sánchez-Infantes[3,5#], Marta Murillo[6#].

[1] Fundació Institut Germans Trias i Pujol, Barcelona, E-08916, Spain.

[2] Centro de Metabolómica y Bioanálisis (CEMBIO), Facultad de Farmacia, Universidad San Pablo-CEU, CEU Universities, Urbanización Montepríncipe, 28660 Boadilla del Monte, Spain.

[3] Department of Basic Health Sciences, Campus Alcorcón, University Rey Juan Carlos (URJC), E-28922 Madrid, Spain.

[4] Departament of Biochemistry and Molecular Biomedicine, and Institut de Biomedicina (IBUB), University of Barcelona, Spain.

[5] Centro de Investigación Biomédica en Red de Fisiopatología de la Obesidad y Nutrición (CIBEROBN), Instituto de Salud Carlos III, E-28029 Madrid, Spain.

[6] Pediatric Department, Hospital Universitari Germans Trias i Pujol, Badalona, E-08916, Spain.

[7] Department of Physical Education, Faculty of Education Sciences, University of Cádiz, Puerto Real, Spain; Biomedical Research Innovation Institute of Cádiz, University of Cádiz, Cádiz, Spain; CIBER of Frailty and Healthy Aging (CIBERFES), Instituto de Salud Carlos III, Madrid, Spain.

*Both authors contributed equally to this work

[#]Co-corresponding

Running title: rhGH treatment effect on lipidome

Keywords: recombinant human growth hormone, lipidome, brown adipose tissue, small gestational age

Contact info: Pediatric Department, Hospital Universitari Germans Trias i Pujol, Badalona, E-08916, Spain.:

mmurillo.germanstrias@gencat.cat

Department of Health Sciences, Campus Alcorcón, University Rey Juan Carlos (URJC), E-28922 Madrid, Spain. Electronic address: david.sanchezi@urjc.es

Supplementary figures

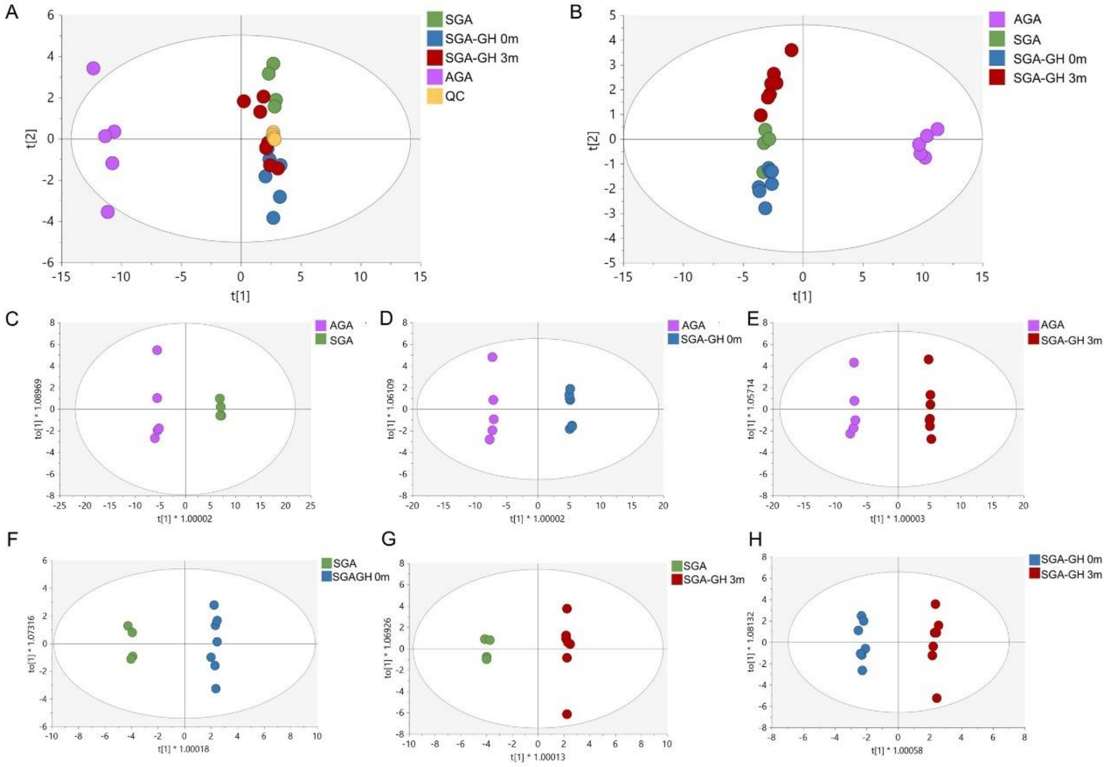

Supplementary Figure S1. **Multivariate plots for statistical analysis**. QC (yellow) are the quality control samples analyzed throughout the analytical run; AGA (purple) corresponds to the average-for-gestational-age group; SGA (green) is the small-for-gestational-age without GH deficiency group; SGA-GH 0m (blue) is the small for gestational age without GH deficiency before rhGH treatment; and SGA-GH 3m (red) is the small for gestational age without GH deficiency after three-month rhGH treatment. Plots A and B represent the PCA and PLS-DA score plots obtained, respectively. As can be observed, the QCs were tightly clustered ensuring the stability and robustness of the analytical performance. The supervised PLS-DA plot displayed a CV-ANOVA *p*-value of 1.2×10^{-7}, an R^2 of 0.977, and a Q^2 of 0.928. C, D, E, F, G, and H are the supervised OPLS-DA plots. Plot C has a CV-ANOVA *p*-value of 0.00062, an R^2 of 0.998 and Q^2 of 0.945. Plot D has a CV-ANOVA *p*-value of 0.0019, an R^2 of 0.999 and a Q^2 of 0.958. Plot E has a CV-ANOVA *p*-value of 0.00032, an R^2 of 0.997 and a Q^2 of 0.921. Plot F has a CV-ANOVA *p*-value of 5.1×10^{-5}, an R^2 of 0.999 and Q^2 of 0.996. Plot G has a CV-ANOVA *p*-value of 2.6×10^{-8}, an R^2 of 0.999 and a Q^2 of 0.997. Plot H has a CV-ANOVA *p*-value of 1.2×10^{-7}, an R^2 of 0.999, and a Q^2 of 0.993.

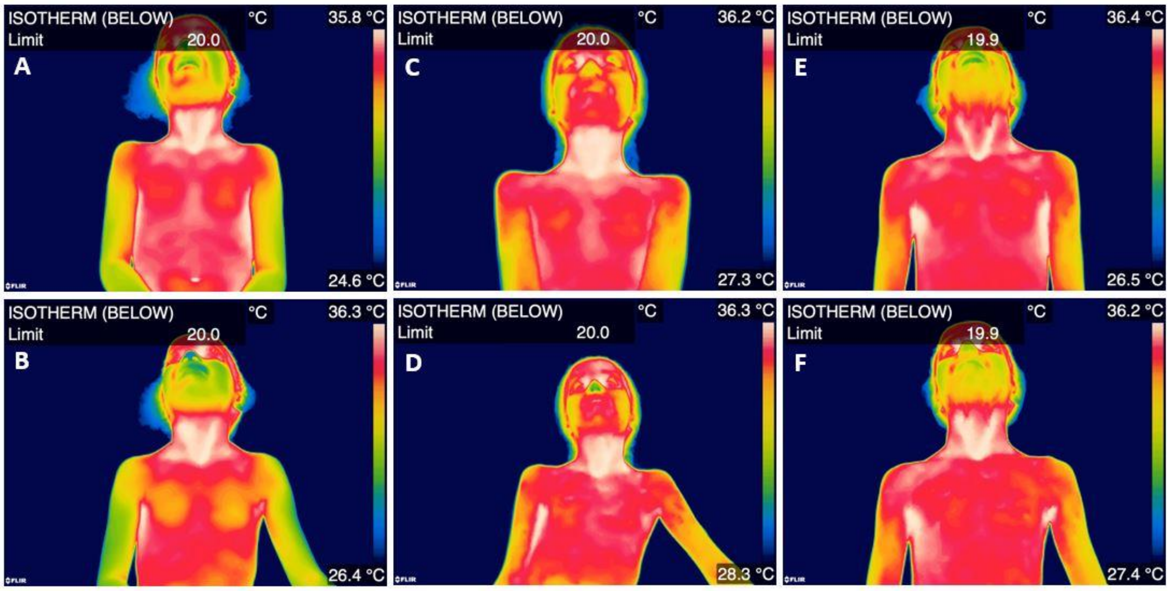

Supplementary Figure S2. Representative IRT images of an SGA child at baseline, 3 and 12 months after rhGH treatment. IRT before rhGH treatment at 0 min (a) and after 5 min (b) of cold exposure. IRT 3 months after rhGH treatment at 0 min (c) and after 5 min (d) of cold exposure. IRT 12 months after rhGH treatment at 0 min (e) and after 5 min (f) of cold exposure.

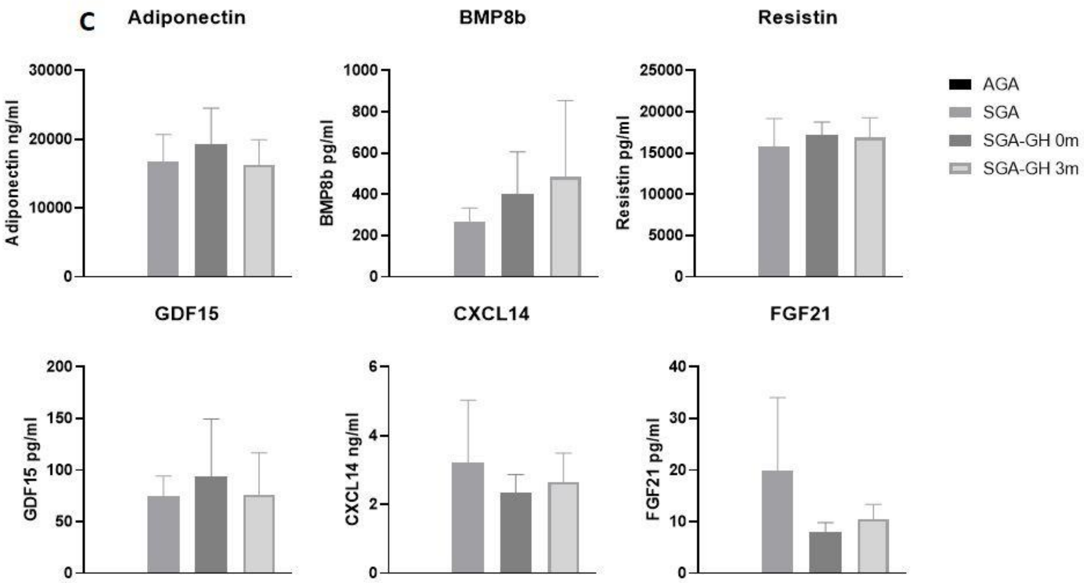

Supplementary Figure S3. Circulating serum levels of metabolites after 3 months of rhGH treatment. Circulating Adiponectin, BMP8b, Resistin, GDF15, CXCL14 and FGF21 levels in AGA and SGA children untreated with rhGH, and rhGH-treated SGA children at baseline and 3 and 12 months after rhGH administration. Data shown are means+/-SD. A two-tailed unpaired Student's t-test was used to compare circulating protein levels in AGA, untreated SGA, and treated SGA groups at baseline. One-

way paired ANOVA was used to compare circulating protein levels in the SGA-GH group at baseline and 3 months after rhGH treatment.

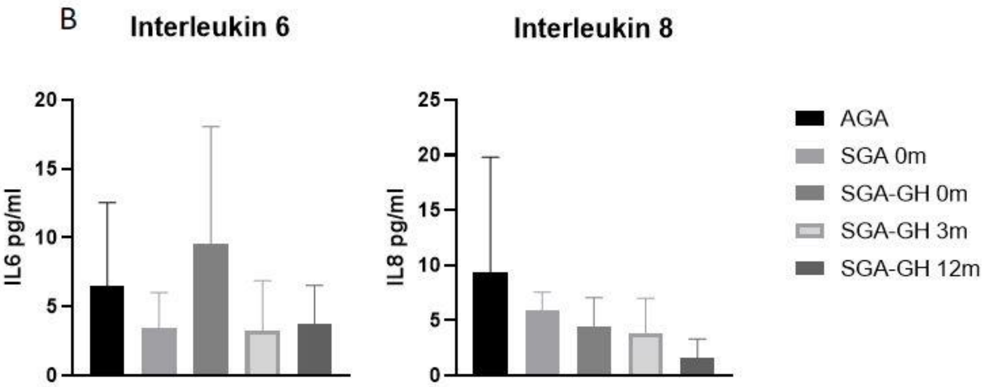

Supplementary Figure S4. Circulating serum levels of metabolites after 3 and 12 months of rhGH treatment. Circulating Interleukin 6 and 8 levels in AGA and SGA children untreated with rhGH, and rhGH-treated SGA children at baseline and 3 and 12 months after rhGH administration. Data shown are means+/-SD. A two-tailed unpaired Student's t-test was used to compare circulating protein levels in AGA, untreated SGA, and rhGH-treated SGA groups at baseline. One-way paired ANOVA was used to compare circulating protein levels in the SGA-GH group at baseline and 3 and 12 months after rhGH treatment.

Supporting Table S1 Clinical and anthropometric data of the study participants. Infants born adequate for gestational age (AGA); infants born small for gestational age (SGA); and infants born small for gestational who undergo GH treatment (SGA-GH). Data shown are means +/- SD.

	AGA	SGA	SGA-GH		
	0M	0M	0M	3M	12M
**Infant data**					
Sex (F/M)	5/0	2/2	6/1		
Multiple pregnancy	-	2	0		
Gestational age (weeks)	38.4 (3.3)	34.9 (1.7)	37.9 (1.6)		
Birth weight (Kg)	3.0 (1.0)	1.7 (0.3)	2.2 (0.6)		
Birth size (cm)	48.6 (5.3)	41.6 (2.4)	43.6 (2.9)		
Age (years)	5.5 (1.6)	6.5 (1.1)	5.1 (1.4)		
Size (cm)	111.2 (8.1)	108.2 (5.5)	97.8 (8.7)	100.5 (8.2)	105 (8.8)
Weight (kg)	20.5 (4.2)	17.4 (3.4)	13.9 (2.5)	14.3 (2.5)	15.6 (2.9)
IGF-1 (SDS)	-	0.2 (0.7)	0.1 (1.2)	1.0 (1.1)	1.0 (0.9)

Supporting Table S2. Summary of SGA and SGA-GH clinical and anthropometric data. Weight, size and IGF1 standard deviation score (SDS) at birth and baseline.

ID	Sex (F/M)	GH Treatment	Birth Size desviation (SDS)	Birth Weight desviation (SDS)	Baseline Age (years)	Baseline Size desviation (SDS)	Target Size (SDS)	Baseline IGF1 Desviation (SDS)
B1	M	No	-2.3	-1.6	6.4	-2.1	-0.6	
B2	F	No	-1.8	-2.7	4.9	-2.1	-0.3	
B3	F	No	-2.0	-1.4	7.2	-2.5	-0.6	
B4	M	No	-2.7	-1.7	7.1	-2.3	-1.7	
C1	M	Yes	-2.9	-2.9	4.6	-2.6	-1.7	+0.2
C2	F	Yes	-3.6	-2.7	4.3	-2.5	-0.7	+0.5
C3	F	Yes	-2.2	-0.6	5.5	-2.5	-1.9	+0.8
C5	F	Yes	-3.2	-2.9	4.8	-3.6	-1.8	-0.1
C6	F	Yes	-2.4	-1.5	4.2	-3.2	-0.6	+1.5
C4	F	Yes	-2.0	-2.4	7.1	-2.8	-0.7	-0.1
C7	F	Yes	-2.0	-1.0	4	-2.7	-0.5	-0.2

Supporting Table S3. Growth parameters evolution in SGA patients treated with GH. Weight, size and IGF1 standard deviation score (SDS) at birth, baseline and after 3 and 12 months of GH treatment. GH treatment adherence data.

ID	Baseline Size Desviation (SDS)	3M Size Desviation (SDS)	12M Size Desviation (SDS)	Groth velocity (baseline -12 moths, cm/year)	Baseline IGF1 Desviation (SDS)	3M IGF1 Desviation (SDS)	12M IGF1 Desviation (SDS)	GH Treatment Adherence (%)
C1	-2.6	-2	-2.3	+3.8	+0.2	+0.2	+1.3	98%
C2	-2.5	-2.1	-2.1	+0.9	+0.5	+2.5	+1.1	100%
C3	-2.5	-2.2	-2	+0.8	+0.8	+2.1	+2.1	100%
C5	-3.6	-3	-3.3	+1.9	-0.1	+0.2	+0.4	94%
C6	-3.2	-3	-2.8	+0.9	+1.5	+1.6	+2	90%
C4	-2.8	-2.5	-2	+2.5	-0.1	-0.2	-0.1	86%
C7	-2.7	-2.7	-3.5	-0.1	-0.2	+0.01	+0.1	89%

Supporting Table S4. Lipids found to be statistically significant for any of the comparisons performed at different SGA stages and GH status.

Mass	*m/z*	RT (min)	Candidate	Formula	Mode	Adduct	Error	Source	CV in QC(%)	SGA-GH0m vs SGA	p value	pBH	VIP	SGA-GH3m vs SGA	p value	pBH	VIP	SGA-GH3m vs SGA-GH0m	p value	pBH	VIP
446.3392	445.332	2.42	(24R)-1α,24-dihydroxy-26,27-dimethyl-22-oxavitamin D3 / (24R)-1α,24-dihydroxy-26,27-dimethyl-22-oxacholecalciferol	C28H46O4	ESI (-)	(M-H)-	1	MS/MS	3.5	-41.0	0.024	0.028	1.2	-21.8	0.170	0.91	0.6	32.5	0.047	0.12	0.9
567.422	568.4292	5.41	1-(2-methoxy-tricosanyl)-sn-glycero-3-phosphoethanolamine	C29H62NO7P	ESI (+)	(M+H)+	8	MS/MS	6.0	75.9	0.024	0.030	1.6	101.9	0.009	0.04	1.3	14.8	0.297	0.46	0.7
468.381	467.3738	1.84	17-oxo-hexacosenoic acid	C26H48O3	ESI (-)	(M+C2H3O2)-	1	MS/MS	10.5	34.8	0.042	0.042	0.7	17.2	0.307	0.99	0.4	-13.1	0.031	0.12	0.6
362.2429	361.2357	2.62	1a,1b-dihomo-PGJ2 / 1a,1b-dihomo-15-deoxy-delta-12,14-PGD2	C22H34O4	ESI (-)	(M-H)-	8	MS	2.9	-14.2	0.527	0.861	0.6	42.8	0.049	0.79	0.8	66.4	0.016	0.12	1.3
340.2406	339.2334	3.21	1-O-(2R-hydroxy-tetradecyl)-sn-glycerol	C17H36O4	ESI (-)	(M+Cl)-	9	MS/MS	0.8	7.2	0.927	0.973	1.8	-17.6	0.341	0.99	0.4	-23.1	0.375	0.53	0.5
869.7568	870.764	13.58	1-O-eicosanoyl-Cer(d18:1/16:0)	C54H105NO4	ESI (+)	(M+K)+	4	MS	7.6	-4.2	0.927	0.987	0.5	35.9	0.346	0.64	0.8	41.9	0.078	0.17	1.0
315.314	316.3212	2.98	6-Hydroxy-4-nonadecanone	C19H38O2	ESI (+)	(M+NH4)+	1	MS	2.6	44.0	0.024	0.030	1.5	-30.6	0.159	0.49	1.8	-51.8	0.016	0.12	2.2
313.2249	314.2321	0.91	ACar 10:1	C17H31NO4	ESI (+)	(M+H)+	0	MS/MS	4.7	-4.8	0.788	0.926	0.4	30.3	0.358	0.96	0.7	36.9	0.078	0.17	1.0
367.2729	368.2801	1.16	ACar 14:2	C21H37NO4	ESI (+)	(M+H)+	1	MS/MS	3.4	-8.5	0.648	0.877	0.4	70.0	0.134	0.49	0.9	85.8	0.031	0.12	1.3
273.2662	274.2734	1.72	C16 Sphinganine	C16H35NO2	ESI (+)	(M+H)+	2	MS/MS	5.6	168.4	0.006	0.010	1.7	-46.7	0.084	0.49	1.3	-80.2	0.063	0.16	2.7
712.633	711.6258	12.52	CE 18:0	C45H80O2	ESI (-)	(M+C2H3O2)-	6	MS/MS	3.5	-29.2	0.020	0.673	1.7	-24.9	0.040	0.79	0.8	6.0	0.156	0.28	0.2
693.6616	692.6544	12.54	Cer 18:0;O/24:1	C42H83NO2	ESI (-)	(M+C2H3O2)-	3	MS/MS	21.1	-36.2	0.010	0.019	1.1	-15.9	0.172	0.91	0.6	31.7	0.109	0.21	1.2
683.6414	682.6342	12.41	Cer 18:0;O2/22:0	C40H81NO3	ESI (-)	(M+C2H3O2)-	1	MS/MS	17.4	-45.7	0.015	0.028	1.2	-38.2	0.015	0.03	1.0	13.8	0.469	0.61	0.6
653.5959	652.5887	11.93	Cer 18:1;O2/20:0	C38H75NO3	ESI (-)	(M+C2H3O2)-	0	MS/MS	2.8	-34.9	0.010	0.019	1.0	-31.8	0.041	0.79	0.9	4.8	0.469	0.61	0.4
621.6043	620.5971	12.21	Cer 18:1;O2/22:0	C40H79NO3	ESI (-)	(M-H)-	1	MS/MS	1.4	-27.4	0.044	0.798	1.7	-19.6	0.246	0.91	0.5	2.5	0.297	0.46	0.4
649.6366	648.6294	12.51	Cer 18:1;O2/24:0	C42H83NO3	ESI (-)	(M-H)-	1	MS/MS	1.3	-30.0	0.023	0.673	3.0	-26.1	0.032	0.79	0.8	5.5	0.297	0.46	0.2
647.6209	646.6137	12.23	Cer 18:1;O2/24:1	C42H81NO3	ESI (-)	(M-H)-	1	MS/MS	1.3	-29.9	0.046	0.673	2.1	-27.6	0.047	0.79	0.8	3.3	0.578	0.68	0.3
761.6891	762.6963	12.23	Cer 20:0;O3/26:0;O	C46H93NO5	ESI (+)	(M+Na)+	2	MS/MS	2.2	-13.5	0.490	0.721	0.4	35.0	0.023	0.04	1.0	56.2	0.016	0.12	1.5
525.512	526.5192	11.85	Cer 18:0;O2/15:0	C33H67NO3	ESI (+)	(M+H)+	0	MS/MS	0.5	-10.2	0.639	1.000	0.9	-15.3	0.469	0.96	1.5	-4.3	0.375	0.53	1.5
1493.1287	1516.1318	8.09	CL(74:0)	C83H162O17P2	ESI (+)	(M+Na)+	10	MS	1.9	-11.2	0.577	0.987	0.6	34.1	0.225	0.58	0.8	51.0	0.047	0.12	1.2
680.5586	679.5514	11.94	DG 16:0/20:2/0:0	C39H72O5	ESI (-)	(M+C2H3O2)-	1	MS/MS	2.0	26.6	0.222	0.861	1.3	-2.8	0.864	0.99	0.2	-23.2	0.156	0.28	0.8
676.5298	675.5226	10.52	DG 18:1/18:3/0:0	C39H68O5	ESI (-)	(M+C2H3O2)-	3	MS/MS	5.9	-43.4	0.008	0.023	1.3	-35.7	0.006	0.02	1.0	13.7	0.297	0.46	0.6
200.1775	199.1703	1.46	FA 12:0 Lauric acid	C12H24O2	ESI (-)	(M-H)-	0	MS/MS	4.2	-46.8	0.002	0.019	1.3	-22.3	0.119	0.91	0.8	46.1	0.047	0.12	1.1
228.2086	227.2014	2.12	FA 14:0 Myristic acid	C14H28O2	ESI (-)	(M-H)-	1	MS/MS	1.5	4.9	0.858	0.973	0.5	55.5	0.030	0.98	1.0	48.2	0.031	0.12	1.2
226.1931	225.1859	1.62	FA 14:1 Myristoleic acid	C14H26O2	ESI (-)	(M-H)-	0	MS/MS	4.6	42.8	0.035	0.673	0.9	68.2	0.011	0.98	1.1	17.8	0.078	0.17	0.7
242.2242	241.217	2.56	FA 15:0	C15H30O2	ESI (-)	(M-H)-	1	MS/MS	17.6	31.2	0.005	0.861	0.9	61.8	0.012	0.73	1.0	31.1	0.109	0.21	0.7
256.2406	255.2334	3.02	FA 16:0 Palmitic acid	C16H32O2	ESI (-)	(M-H)-	2	MS/MS	1.4	-30.9	0.008	0.861	1.0	-25.8	0.037	0.99	0.9	7.3	0.578	0.68	0.6
254.2243	253.2171	2.33	FA 16:1 Palmitoleic acid	C16H30O2	ESI (-)	(M-H)-	1	MS/MS	1.3	-5.1	0.634	0.904	0.2	20.2	0.335	0.99	0.6	26.7	0.109	0.21	0.8
278.2241	277.2169	2.12	FA 18:3 alpha-Linolenic acid	C18H30O2	ESI (-)	(M-H)-	1	MS/MS	1.0	-37.9	0.006	0.023	1.1	-22.9	0.192	0.98	0.7	24.0	0.375	0.53	0.6
313.2981	314.3053	4.07	FA 19:1	C19H36O2	ESI (+)	(M+NH4)+	1	MS/MS	1.8	39.1	0.041	0.635	0.8	54.6	0.041	0.04	1.0	15.2	0.578	0.68	0.7
304.24	303.2328	2.54	FA 20:4 Arachidonic acid	C20H32O2	ESI (-)	(M-H)-	1	MS/MS	2.2	47.3	0.010	1.000	1.0	59.1	0.004	0.99	1.1	8.1	0.375	0.53	0.6
332.2718	331.2646	3.27	FA 22:4 Adrenic acid	C22H36O2	ESI (-)	(M-H)-	1	MS/MS	2.8	-12.6	0.480	0.904	0.7	28.9	0.307	0.99	0.6	47.5	0.016	0.12	1.0
330.2558	329.2486	2.76	FA 22:5 DPA	C22H34O2	ESI (-)	(M-H)-	0	MS/MS	1.2	12.6	0.656	0.973	0.2	36.4	0.233	0.99	0.7	21.1	0.375	0.53	0.7
354.3493	353.3421	5.69	FA 23:0	C23H46O2	ESI (-)	(M-H)-	1	MS	7.8	-24.4	0.199	0.857	0.7	-33.2	0.050	0.04	0.9	-11.6	0.578	0.68	0.5
440.3862	439.379	5.08	FA 25:1	C25H48O2	ESI (-)	(M+C2H3O2)-	1	MS/MS	5.2	42.2	0.005	0.023	1.0	-27.0	0.131	0.98	0.8	-48.7	0.016	0.12	1.6
396.396	395.3888	7.94	FA 26:0	C26H52O2	ESI (-)	(M-H)-	2	MS/MS	8.6	-33.9	0.009	0.023	1.0	-29.4	0.020	0.03	0.8	6.9	0.375	0.53	0.3
412.3908	411.3836	4.16	FA 26:0;O	C26H52O3	ESI (-)	(M-H)-	2	MS	2.4	-23.3	0.020	0.673	0.7	-39.0	0.004	0.03	1.1	-20.5	0.109	0.21	0.8
280.2408	279.2336	2.62	FA 18:1;O	C18H34O3	ESI (-)	(M-H-H2O)-	4	MS/MS	0.6	-19.0	0.160	0.857	1.3	44.6	0.010	0.99	0.9	78.5	0.016	0.12	4.5

665.6269	666.6341	12.12	FAHFA(22:1-(18-O-20:0))	C42H80O4	ESI (+)	(M+H4N)+	8	MS/MS	3.7	590.7	0.006	0.010	2.2	777.8	0.013	0.02	2.2	27.1	0.469	0.61	0.6
449.3141	448.3069	0.9	Glycoursodeoxycholic acid	C26H43NO5	ESI (-)	(M-H)-	0	MS/MS	3.0	34.6	0.162	0.798	0.6	-33.7	0.034	0.79	1.0	-50.7	0.031	0.12	1.6
783.6609	784.6681	12	HexCer 18:1/22:0	C46H89NO8	ESI (+)	(M+H)+	5	MS/MS	3.9	-52.2	0.034	0.541	1.3	-50.2	0.040	0.49	1.5	-7.4	0.938	0.95	1.0
837.7089	855.7426	13.96	HexCer 18:1/26:1	C50H95NO8	ESI (+)	(M+NH4)+	4	MS/MS	5.7	-20.0	0.377	0.804	0.9	18.4	0.430	0.64	0.7	48.0	0.031	0.12	1.3
763.6287	762.6215	12.51	HexCer 18:2;O2/22:0	C46H87NO8	ESI (-)	(M-H-H2O)-	4	MS/MS	3.2	-24.2	0.039	0.042	0.8	-20.1	0.111	0.91	0.6	5.5	0.375	0.53	0.2
873.6555	872.6483	11.54	Hex2Cer 18:0;O2/18:0	C48H93NO13	ESI (-)	(M+C2H3O2)-	2	MS	13.3	-35.8	0.014	0.028	1.0	-32.6	0.009	0.03	0.9	5.0	0.813	0.86	0.2
161.105	162.1122	0.78	L-carnitine	C7H16NO3	ESI (+)	(M+H)+	2	MS/MS	1.7	-27.6	0.078	0.635	1.2	-29.2	0.047	0.04	0.8	-2.1	0.813	0.86	0.4
541.3182	542.3254	1.75	LPC 0:0/20:5	C28H48NO7P	ESI (+)	(M+H)+	1	MS/MS	7.5	54.1	0.456	0.770	1.4	27.5	0.516	0.64	0.9	-17.3	0.578	0.68	0.9
481.3164	482.3236	2.18	LPC 15:0/0:0	C23H48NO7P	ESI (+)	(M+H)+	0	MS/MS	4.9	17.1	0.449	0.926	0.4	63.7	0.038	0.49	1.1	27.5	0.031	0.12	1.0
509.348	510.3552	3.04	LPC 17:0/0:0	C25H52NO7P	ESI (+)	(M+H)+	0	MS/MS	3.0	38.3	0.031	0.987	1.2	87.0	0.045	0.58	1.2	36.1	0.109	0.21	1.0
519.3327	520.3399	2.23	LPC 18:2/0:0	C26H50NO7P	ESI (+)	(M+H)+	1	MS/MS	10.8	-7.8	0.709	0.721	0.7	22.2	0.445	0.59	0.7	32.6	0.219	0.35	1.0
517.3163	518.3235	1.81	LPC 18:3/0:0	C26H48NO7P	ESI (+)	(M+H)+	1	MS/MS	8.2	10.5	0.607	1.000	0.2	49.5	0.201	0.42	0.8	35.2	0.219	0.35	1.0
567.3329	568.3401	2.09	LPC 22:6/0:0	C30H50NO7P	ESI (+)	(M+H)+	0	MS/MS	15.5	-12.1	0.587	0.926	0.7	34.7	0.330	0.80	0.7	53.2	0.219	0.35	1.1
270.2555	269.2483	3.49	Methyl hexadecanoate	C17H34O2	ESI (-)	(M-H)-	1	MS	11.7	-22.3	0.108	0.798	0.6	53.8	0.043	0.73	1.1	98.0	0.016	0.12	1.6
390.2981	389.2909	3.39	MG 16:0/0:0/0:0	C19H38O4	ESI (-)	(M+C2H3O2)-	0	MS/MS	3.6	17.8	0.490	0.861	0.3	102.4	0.046	0.03	1.3	71.9	0.109	0.21	1.3
418.3292	417.322	4.31	MG 18:0/0:0/0:0	C21H42O4	ESI (-)	(M+C2H3O2)-	0	MS/MS	2.7	2.9	0.915	0.861	0.9	-22.4	0.156	0.99	0.6	-24.6	0.219	0.35	1.0
788.5216	787.5144	2.56	OxPG 16:0_20:3+1O	C42H77O11P	ESI (-)	(M-H)-	2	MS/MS	6.5	21.8	0.186	0.920	0.5	62.7	0.013	0.98	1.0	33.6	0.078	0.17	1.1
764.5231	763.5159	2.6	OxPG 16:0_16:1+1O	C40H77N2O7P	ESI (-)	(M-H)-	0	MS/MS	5.8	-38.1	0.018	0.028	1.0	0.6	0.966	1.00	0.2	62.6	0.016	0.12	1.3
745.558	746.5652	9.81	PA 22:2/16:0	C41H77O8P	ESI (+)	(M+NH4)+	6	MS/MS	7.9	118.1	0.012	0.010	1.9	90.7	0.015	0.04	1.1	-12.6	0.688	0.77	0.9
805.5632	806.5704	7.55	PC 16:0/22:6	C46H80NO8P	ESI (+)	(M+H)+	1	MS/MS	2.1	-13.2	0.590	0.877	1.0	15.6	0.632	0.72	0.7	33.2	0.156	0.28	1.1
865.5826	864.5754	7.34	PC 16:0_22:6	C46H80NO8P	ESI (-)	(M+C2H3O2)-	0	MS/MS	2.2	-27.1	0.074	0.861	1.3	6.0	0.799	0.99	0.4	45.5	0.016	0.12	1.1
779.5464	780.5536	6.9	PC 16:0/20:5	C44H78NO8P	ESI (+)	(M+H)+	0	MS/MS	2.9	41.3	0.397	0.804	0.5	81.4	0.136	0.49	1.1	28.4	0.813	0.86	0.9
815.566	814.5588	6.61	PC 16:1/18:2	C44H82NO10P	ESI (-)	(M-H)-	2	MS/MS	5.6	-37.1	0.001	0.019	1.1	12.2	0.339	0.99	0.5	78.3	0.016	0.12	1.5
783.5784	784.5856	8.34	PC 18:1/18:2	C44H82NO8P	ESI (+)	(M+H)+	1	MS/MS	2.9	-8.0	0.603	1.000	0.5	20.0	0.477	0.80	0.7	30.5	0.219	0.35	1.0
781.561	782.5682	7.12	PC 18:2/18:2	C44H80NO8P	ESI (+)	(M+H)+	1	MS/MS	1.3	-20.6	0.143	0.541	1.0	32.9	0.333	0.59	0.7	67.3	0.047	0.12	1.3
779.5445	780.5517	6.6	PC 18:2/18:3	C44H78NO8P	ESI (+)	(M+H)+	1	MS/MS	2.0	-4.9	0.701	0.987	0.1	29.6	0.286	0.59	0.8	36.3	0.047	0.12	1.0
771.5781	772.5853	9.11	PC 17:0_18:2	C43H82NO8P	ESI (+)	(M+H)+	0	MS/MS	2.6	3.5	0.863	0.987	0.1	53.8	0.038	0.49	1.2	31.1	0.078	0.17	1.2
819.5793	820.5865	8.44	PC 17:1_22:5	C47H82NO8P	ESI (+)	(M+H)+	0	MS/MS	3.9	36.2	0.123	0.877	0.8	71.4	0.037	1.24	1.1	43.8	0.375	0.53	0.7
805.5622	806.5694	6.93	PC 18:2/20:4	C46H80NO8P	ESI (+)	(M+H)+	0	MS/MS	2.7	-19.3	0.019	0.381	0.7	15.1	0.547	0.59	0.7	42.8	0.078	0.17	1.0
831.5779	832.5851	7.77	PC 40:7	C48H82NO8P	ESI (+)	(M+H)+	1	MS/MS	3.7	-11.2	0.623	1.000	0.7	6.8	0.849	0.80	0.9	20.3	0.578	0.68	1.1
819.5791	820.5863	8.14	PC 17:0/22:6	C47H82NO8P	ESI (+)	(M+H)+	0	MS/MS	5.2	64.1	0.026	0.987	1.1	96.9	0.015	0.72	1.2	59.4	0.578	0.68	0.7
795.5777	796.5849	8.53	PC 17:0/20:4	C45H82NO8P	ESI (+)	(M+H)+	1	MS/MS	3.5	76.0	0.023	0.770	1.1	80.2	0.005	0.58	1.2	29.0	0.938	0.95	0.7
779.5451	780.5523	6.27	PC 18:1_18:4	C44H78NO8P	ESI (+)	(M+H)+	1	MS/MS	3.0	-17.0	0.335	0.721	0.5	66.8	0.122	0.49	0.8	100.9	0.047	0.12	1.4
743.5458	744.553	7.25	PC 15:0/18:2	C41H78NO8P	ESI (+)	(M+H)+	0	MS/MS	2.6	10.3	0.594	0.804	0.2	59.6	0.002	0.42	1.1	40.1	0.031	0.12	1.1
719.5462	720.5534	8.22	PC 16:0/15:0	C39H78NO8P	ESI (+)	(M+H)+	0	MS/MS	1.1	68.6	0.014	0.635	1.1	73.0	0.019	0.49	1.1	23.3	0.813	0.86	0.7
771.5784	772.5856	8.77	PC 17:1_18:1	C43H82NO8P	ESI (+)	(M+H)+	0	MS/MS	3.3	58.5	0.007	0.770	1.1	56.0	0.013	0.49	1.0	35.0	0.938	0.95	0.4
773.5945	774.6017	10.47	PC 17:0/18:1	C43H84NO8P	ESI (+)	(M+H)+	0	MS/MS	2.7	99.7	0.030	0.770	1.4	84.1	0.013	0.59	1.2	19.9	0.469	0.61	0.6
729.5306	730.5378	6.54	PC 14:0/18:2	C40H76NO8P	ESI (+)	(M+H)+	0	MS/MS	2.7	-14.8	0.393	0.635	0.5	25.2	0.308	0.88	0.6	46.9	0.109	0.21	1.0
769.5609	770.5681	7.72	PC 35:3	C43H80NO8P	ESI (+)	(M+H)+	2	MS/MS	16.2	26.6	0.327	0.721	0.5	53.9	0.018	0.04	1.2	21.6	0.469	0.61	0.9
783.5768	784.584	9.25	PC 16:0/20:3	C44H82NO8P	ESI (+)	(M+H)+	1	MS/MS	3.2	97.0	0.070	0.030	1.9	54.1	0.208	0.58	0.6	-21.8	0.297	0.46	0.8
831.5779	832.5851	7.35	PC 18:2/22:5	C48H82NO8P	ESI (+)	(M+H)+	1	MS/MS	4.7	-33.7	0.008	0.030	1.0	-12.4	0.580	0.80	0.7	32.2	0.156	0.28	0.8
767.5444	768.5516	7.07	PC 15:0/20:4	C43H78NO8P	ESI (+)	(M+H)+	2	MS/MS	6.8	37.0	0.152	0.635	0.7	65.6	0.015	0.04	1.1	37.9	0.156	0.28	0.9
729.5725	730.5797	8.32	PC 15:0/16:0	C39H78NO8P	ESI (+)	(M+H)+	0	MS/MS	5.6	54.6	0.025	0.877	1.0	116.4	0.0000023	0.75	1.5	8.6	0.047	0.12	1.1
797.5947	798.6019	9.4	PC 19:1/18:2	C45H84NO8P	ESI (+)	(M+H)+	1	MS/MS	2.9	28.6	0.173	0.877	0.5	37.5	0.014	0.58	1.0	28.4	0.578	0.68	0.6
703.5119	704.5191	6.22	PC 16:0/14:1	C38H74NO8P	ESI (+)	(M+H)+	5	MS/MS	8.4	227.2	0.012	0.010	2.7	226.2	0.014	0.02	1.5	-0.3	0.938	0.95	0.8
719.5466	720.5538	7.9	PC 18:0/13:0	C39H78NO8P	ESI (+)	(M+H)+	0	MS/MS	1.7	61.4	0.166	0.502	1.2	40.1	0.399	0.64	0.6	-13.2	1.000	1.00	0.8
791.5514	792.5586	6.79	PC 37:6	C45H78NO8P	ESI (+)	(M+H)+	3	MS/MS	2.3	3.4	0.911	0.987	0.3	80.7	0.130	0.49	1.1	74.8	0.016	0.12	1.5
777.5395	778.5467	6.17	PC 36:6	C44H76NO8P	ESI (+)	(M+H)+	7	MS/MS	5.7	-34.0	0.040	0.489	1.3	12.1	0.730	0.88	0.8	69.8	0.078	0.17	1.1

799.611	800.6182	11.18	PC 19:0/18:2	C45H86NO8P	ESI (+)	(M+H)+	0	MS/MS	7.1	-49.9	0.113	0.381	1.1	-20.9	0.592	0.72	0.8	57.8	0.375	0.53	0.9
831.5767	832.5839	7.96	PC 20:4/20:3	C48H82NO8P	ESI (+)	(M+H)+	1	MS/MS	19.4	-3.9	0.903	0.987	0.5	61.9	0.334	0.72	1.0	68.4	0.156	0.28	1.4
807.5781	808.5853	8.34	PC 38:5	C46H82NO8P	ESI (+)	(M+H)+	2	MS/MS	4.1	4.2	0.834	1.000	0.1	35.7	0.261	0.64	0.8	30.3	0.109	0.21	1.0
779.5462	780.5534	7.38	PC 16:1_20:4	C44H78NO8P	ESI (+)	(M+H)+	0	MS/MS	18.4	101.9	0.006	0.010	1.8	59.9	0.179	0.64	0.8	-20.8	0.688	0.77	0.8
765.555	766.5622	7.87	PC 18:3/P-18:1	C44H80NO7P	ESI (+)	(M+H)+	1	MS/MS	1.4	287.8	0.026	0.010	1.9	7.7	0.820	0.96	0.5	-72.2	0.016	0.12	2.5
757.5623	758.5695	7.86	PC 16:1/18:1	C42H80NO8P	ESI (+)	(M+H)+	0	MS/MS	2.9	-9.2	0.624	0.926	0.2	26.3	0.335	0.64	0.7	39.1	0.078	0.17	1.0
715.5462	716.5534	8.52	PC O-14:0/18:2	C40H78NO7P	ESI (+)	(M+H)+	8	MS/MS	26.9	65.9	0.091	0.541	1.4	52.5	0.010	0.04	1.0	-8.1	0.578	0.68	0.3
741.5683	742.5755	9.07	PC O-16:1/18:2	C42H80NO7P	ESI (+)	(M+H)+	1	MS/MS	3.2	-29.2	0.003	0.010	1.3	8.0	0.654	0.96	0.4	52.5	0.016	0.12	1.3
793.598	794.6052	10.56	PC O-18:0/20:5	C46H84NO7P	ESI (+)	(M+H)+	1	MS/MS	4.0	-13.5	0.324	0.804	0.6	11.3	0.409	0.64	0.5	28.6	0.047	0.12	0.9
819.6137	820.6209	9.84	PC O-18:0/22:6	C48H86NO7P	ESI (+)	(M+H)+	1	MS/MS	7.9	88.5	0.015	0.042	1.5	62.1	0.144	0.49	0.8	-14.0	0.813	0.86	0.6
797.6306	798.6378	11.58	PC P-16:0/22:2	C46H88NO7P	ESI (+)	(M+H)+	1	MS/MS	7.5	-89.6	0.000	0.010	3.6	-89.7	0.000	0.02	2.5	-0.7	0.813	0.86	0.6
755.5898	756.597	10.28	PC P-18:0/17:2	C43H82NO7P	ESI (+)	(M+H)+	9	MS/MS	7.6	-17.8	0.133	0.804	0.5	24.5	0.361	0.88	0.5	51.5	0.031	0.12	1.0
763.5204	764.5276	7.82	PE 16:0/22:6	C43H74NO8P	ESI (+)	(M+H)+	0	MS/MS	20.7	517.2	0.000	0.010	2.3	50.6	0.182	0.59	0.8	-75.6	0.016	0.12	2.8
745.5606	746.5678	8.52	PE 18:0_18:1	C41H80NO8P	ESI (+)	(M+H)+	2	MS/MS	3.1	18.0	0.577	0.804	0.5	37.6	0.338	0.49	0.8	16.6	0.578	0.68	0.9
767.5462	768.5534	10.47	PE 18:0_20:4	C43H74NO8P	ESI (+)	(M+H)+	1	MS/MS	4.1	-27.7	0.052	0.541	1.3	-23.4	0.014	0.04	1.1	6.0	0.578	0.68	0.6
767.5456	766.5384	10.09	PE 18:1/20:3	C43H78NO8P	ESI (-)	(M-H)-	1	MS/MS	3.3	-20.7	0.209	0.861	0.8	-24.7	0.016	0.03	1.0	-5.1	0.688	0.77	0.3
753.5667	752.5595	11.46	PE O-18:0/20:4	C43H80NO7P	ESI (-)	(M-H)-	1	MS/MS	24.9	-13.8	0.506	0.861	0.6	74.8	0.011	0.03	1.1	102.8	0.016	0.12	1.6
747.5192	746.512	8.47	PE P-16:0/22:6	C43H74NO7P	ESI (-)	(M-H)-	1	MS/MS	3.0	-39.1	0.002	0.019	1.1	-7.1	0.741	0.99	0.4	52.5	0.016	0.12	0.9
727.5501	726.5429	11.28	PE P-18:0/18:2	C41H78NO7P	ESI (-)	(M-H)-	2	MS/MS	1.9	-25.1	0.032	0.028	0.8	-49.2	0.001	0.02	1.3	-32.2	0.031	0.12	1.1
751.5509	750.5437	11.28	PE P-18:0/20:4	C43H78NO7P	ESI (-)	(M-H)-	0	MS/MS	1.6	-23.6	0.113	0.798	1.3	-20.5	0.238	0.98	0.8	4.0	0.688	0.77	0.4
749.5355	750.5427	9.1	PE P-18:0/20:5	C43H76NO7P	ESI (+)	(M+H)+	1	MS/MS	3.4	-24.2	0.232	0.635	0.7	123.8	0.014	0.02	1.4	195.1	0.016	0.12	2.4
760.5302	778.5639	11.52	PG 20:1/15:1	C41H77O10P	ESI (+)	(M+H4N)+	6	MS/MS	6.7	84.8	0.039	0.381	1.2	69.5	0.002	0.96	1.1	-23.6	0.938	0.95	0.3
862.5569	861.5497	7.24	PI 18:2/18:0	C45H83O13P	ESI (-)	(M-H)-	0	MS/MS	1.6	-41.0	0.008	0.028	1.2	-40.2	0.005	0.03	1.1	1.2	0.813	0.86	0.6
775.4387	774.4315	12.01	PS 18:4/18:4	C42H66NO10P	ESI (-)	(M-H)-	5	MS/MS	8.5	34.7	0.168	0.861	0.7	86.1	0.001	0.02	1.2	38.2	0.219	0.35	0.9
839.5661	838.5589	6.73	PS 40:4	C46H82NO10P	ESI (-)	(M-H)-	2	MS	3.5	65.2	0.023	0.023	1.1	74.8	0.038	0.73	1.0	5.8	0.813	0.86	0.6
702.567	703.5742	7.09	SM 18:1;O2/16:0	C39H79N2O6P	ESI (+)	(M+H)+	1	MS/MS	3.0	-99.4	0.000	0.010	4.0	-99.3	0.000	0.02	3.7	18.7	0.219	0.35	0.7
858.6803	857.6731	11.73	SM 17:1;O2/24:1	C46H91N2O6P	ESI (-)	(M+C2H3O2)-	1	MS/MS	4.5	50.0	0.006	0.023	1.0	42.3	0.024	0.91	0.8	-5.1	0.688	0.77	0.4
840.7088	841.716	12.25	SM 18:1;O2/26:1	C49H97N2O6P	ESI (+)	(M+H)+	0	MS/MS	11.7	-26.7	0.025	0.042	1.3	-28.5	0.013	0.96	1.3	28.7	1.000	1.00	0.1
844.6654	843.6582	11.38	SM 18:2;O2/22:0	C45H89N2O6P	ESI (-)	(M+C2H3O2)-	2	MS/MS	4.1	41.2	0.049	0.798	0.9	50.0	0.006	0.04	1.0	5.9	0.469	0.61	0.5
716.5819	717.5891	7.86	SM 18:1;O2/17:0	C40H81N2O6P	ESI (+)	(M+H)+	1	MS/MS	2.6	113.9	0.001	0.804	1.5	119.5	0.037	0.49	1.3	17.2	1.000	1.00	0.6
786.6529	787.6601	11.63	SM 18:1;O2/22:0	C45H91N2O6P	ESI (+)	(M+H)+	2	MS/MS	2.9	-40.1	0.049	0.541	1.0	-7.8	0.463	0.88	0.4	32.4	0.078	0.17	1.3
874.7136	873.7064	12.19	SM 18:1;O2/24:0	C47H95N2O6P	ESI (-)	(M+C2H3O2)-	1	MS/MS	1.3	-19.7	0.022	0.798	1.3	-17.9	0.029	0.99	0.6	2.4	0.688	0.77	0.2
378.3141	377.3069	4.31	ST 24:0;O3	C24H42O3	ESI (-)	(M-H)-	2	MS	5.2	24.1	0.101	0.798	0.7	-11.0	0.488	0.99	0.3	-28.3	0.031	0.12	1.1
949.9006	950.9078	14.03	TG 18:0/18:0/21:0	C60H116O6	ESI (+)	(M+NH4)+	3	MS/MS	12.7	-50.9	0.013	0.042	1.3	-45.4	0.014	0.04	1.2	4.3	0.469	0.61	0.8
871.7619	872.7691	14.23	TG 16:0/18:2/18:2	C55H98O6	ESI (+)	(M+NH4)+	1	MS/MS	8.7	-65.9	0.000	0.926	1.7	-68.1	0.000	0.88	1.8	-20.3	0.469	0.61	0.6
842.6768	843.684	14.49	TG 48:1	C51H96O6	ESI (+)	(M+K)+	0	MS	7.0	119.7	0.164	0.541	1.2	18.1	0.164	0.49	0.6	-46.2	0.578	0.68	0.9
828.7172	829.7244	15.06	TG 48:0	C51H98O6	ESI (+)	(M+H4N)+	1	MS/MS	10.3	31.2	0.315	0.721	1.0	31.2	0.315	0.59	0.7	0.0	0.938	0.95	0.5
981.8709	982.8781	14.84	TG 18:0/20:0/22:5	C63H112O6	ESI (+)	(M+H4N)+	2	MS/MS	5.2	38.2	0.073	0.381	1.2	2.7	0.788	0.88	0.4	-25.7	0.047	0.12	1.0
930.7654	931.7726	14.83	TG 56:5	C59H104O6	ESI (+)	(M+Na)+	1	MS/MS	3.4	-51.9	0.041	0.635	1.2	-49.8	0.047	0.58	1.1	4.7	0.469	0.61	0.3
964.6995	965.7067	13.26	TG 58:10	C61H98O6	ESI (+)	(M+K)+	8	MS	7.8	25.6	1.000	1.000	0.7	109.0	0.024	0.04	1.3	66.3	0.031	0.12	1.6
900.7174	901.7246	13.58	TG 16:0/18:2/20:4	C57H98O6	ESI (+)	(M+Na)+	0	MS/MS	2.8	8.8	0.927	0.987	0.3	77.0	0.648	0.80	1.0	62.8	0.156	0.28	1.2
925.8054	881.7002	13.51	TG 18:0/16:1/17:2	C54H98O6	ESI (+)	(M+K)+	1	MS/MS	6.6	3.7	1.000	1.000	0.1	24.0	0.315	0.59	0.6	19.6	0.031	0.12	1.0
898.7035	899.7107	13.3	TG 54:7	C57H96O6	ESI (+)	(M+Na)+	1	MS	4.2	-17.5	0.230	0.635	1.3	122.3	0.006	0.02	1.2	169.5	0.016	0.12	2.2
890.6711	891.6783	13.58	TG 18:2/16:1/18:2	C55H96O6	ESI (+)	(M+K)+	7	MS/MS	3.4	-15.8	0.788	0.926	1.3	28.7	0.315	0.59	0.8	52.8	0.031	0.12	1.6
927.8257	928.8329	15.56	TG 18:1/18:1/20:2	C59H106O6	ESI (+)	(M+H4N)+	1	MS/MS	19.1	-50.6	0.001	0.541	1.4	-25.8	0.007	0.96	0.8	33.7	0.078	0.17	1.2
977.843	978.8502	13.98	TG 18:4/20:1/22:2	C63H108O6	ESI (+)	(M+NH4)+	2	MS/MS	9.1	137.4	0.006	0.010	1.5	-2.5	1.000	1.00	0.5	-58.9	0.016	0.12	2.1
865.8081	866.8153	16.26	TG 16:0/17:0/18:0	C54H104O6	ESI (+)	(M+H4N)+	2	MS/MS	25.2	56.8	0.788	0.926	1.2	87.0	0.073	0.42	1.1	19.3	0.469	0.61	0.8
834.7676	857.7529	15.82	TG 13:0/16:0/21:0	C53H102O6	ESI (+)	(M+Na)+	5	MS/MS	24.6	47.9	0.109	0.489	1.3	1.000	1.00	0.8	0.4	-28.6	0.219	0.35	0.8

767.6995	768.7067	13.75	TG 14:0/14:0/16:0	C47H90O6	ESI (+)	(M+H4N)+	1	MS/MS	7.9	45.0	0.412	0.770	1.1	14.3	0.788	0.88	0.6	-21.2	0.469	0.61	0.6
916.6918	917.699	13.78	TG 16:1/18:2/20:3	C57H98O6	ESI (+)	(M+K)+	1	MS/MS	4.3	40.8	0.042	0.042	1.2	14.1	0.230	0.58	0.6	-19.0	0.109	0.21	0.7
939.8169	940.8241	13.73	TG 17:0/18:0/22:5	C60H106O6	ESI (+)	(M+NH4)+	9	MS/MS	12.0	6.6	1.000	1.000	0.4	30.5	0.230	0.58	0.8	22.5	0.031	0.12	0.9
979.8555	913.797	13.98	TG 14:1/22:0/o-18:0	C57H110O5	ESI (+)	(M+K)+	2	MS/MS	18.5	-37.4	0.002	0.770	1.1	-42.6	0.002	0.80	1.2	52.4	0.813	0.86	1.3
837.7779	838.7851	15.26	TG 16:0/16:0/17:0	C52H100O6	ESI (+)	(M+H4N)+	1	MS/MS	26.3	23.8	0.527	0.804	0.5	42.8	0.315	0.59	0.7	15.4	0.219	0.35	0.6
927.8248	928.832	15.2	TG 18:1/18:1/20:2	C59H106O6	ESI (+)	(M+H4N)+	1	MS/MS	10.7	-52.3	0.023	0.381	1.4	-50.1	0.038	0.04	1.3	17.8	0.578	0.68	0.9
845.7463	846.7535	13.1	TG 14:0/18:1/18:2	C53H96O6	ESI (+)	(M+H4N)+	0	MS/MS	22.5	42.3	0.109	0.489	1.1	44.8	0.014	0.04	0.9	1.7	0.688	0.77	0.7
817.7158	818.723	13.55	TG 48:3	C51H92O6	ESI (+)	(M+H4N)+	1	MS/MS	13.1	22.6	1.000	1.000	0.5	-7.7	0.788	0.88	0.7	-24.7	0.219	0.35	1.0
923.7856	924.7928	13.08	TG 18:0/18:1/20:5	C59H102O6	ESI (+)	(M+NH4)+	10	MS/MS	10.8	-3.2	0.648	0.877	0.5	17.3	0.527	0.72	0.7	21.2	0.016	0.12	0.9
925.807	926.8142	14.54	TG 18:1/18:1/20:3	C59H104O6	ESI (+)	(M+H4N)+	2	MS/MS	9.3	39.8	0.788	0.926	0.2	120.6	0.412	0.64	1.3	57.8	0.219	0.35	1.3
843.7309	844.7381	13.51	TG 14:0/18:2/18:2	C53H94O6	ESI (+)	(M+H4N)+	1	MS/MS	10.4	-1.0	0.788	0.926	0.5	25.8	0.230	0.58	0.8	27.1	0.078	0.17	0.9
927.8251	928.8323	13.91	TG 18:0/18:3/20:1	C59H106O6	ESI (+)	(M+NH4)+	1	MS/MS	5.4	-8.8	0.527	0.804	0.4	13.4	0.788	0.88	0.5	24.4	0.016	0.12	0.9
929.8403	930.8475	15.74	TG 18:1/18:1/20:1	C59H108O6	ESI (+)	(M+H4N)+	1	MS/MS	10.3	-35.8	0.017	0.721	1.1	-38.6	0.000	0.42	1.1	-3.6	0.938	0.95	0.2
1033.9046	1034.9118	14.65	TG 20:0/22:1/22:6	C67H116O6	ESI (+)	(M+H4N)+	1	MS/MS	17.3	-36.7	0.030	0.381	1.1	-39.0	0.010	1.00	1.0	-8.2	0.813	0.86	0.3
899.7944	900.8016	14.45	TG 18:1/18:1/18:2	C57H102O6	ESI (+)	(M+H4N)+	1	MS/MS	7.5	-53.6	0.022	0.877	1.3	-45.3	0.047	1.00	1.0	-6.4	0.031	0.12	0.7
871.7635	872.7707	13.96	TG 16:1/18:1/18:2	C55H98O6	ESI (+)	(M+H4N)+	1	MS/MS	8.0	-20.0	0.374	0.804	1.0	20.4	0.315	0.59	0.8	50.5	0.047	0.12	1.3
912.7898	952.8277	13.96	TG 18:0/20:1/20:5	C61H106O6	ESI (+)	(M+NH4)+	5	MS/MS	5.6	-21.0	0.293	0.770	0.5	30.7	0.315	0.59	0.8	65.4	0.016	0.12	1.2
901.8102	902.8174	15.03	TG 18:1/18:1/18:1	C57H104O6	ESI (+)	(M+H4N)+	1	MS/MS	7.5	-49.8	0.009	0.877	1.3	-45.1	0.017	0.64	1.2	50.0	0.688	0.77	0.6
925.8081	926.8153	14.66	TG 16:0/18:1/22:4	C59H104O6	ESI (+)	(M+H4N)+	1	MS/MS	8.6	9.6	0.527	0.804	0.4	44.1	0.042	0.04	0.8	31.5	0.109	0.21	0.8
936.8146	954.8482	13.96	TG 58:5	C61H108O6	ESI (+)	(M+NH4)+	0	MS	5.6	-17.9	0.412	0.770	1.1	17.9	0.315	0.59	0.7	43.6	0.047	0.12	1.0
897.7786	898.7858	13.98	TG 18:1/18:2/18:2	C57H100O6	ESI (+)	(M+H4N)+	1	MS/MS	8.1	-19.7	0.648	0.877	1.0	26.0	0.527	0.72	0.8	57.1	0.031	0.12	1.4
925.8091	926.8163	14.83	TG 18:0/18:1/20:4	C59H104O6	ESI (+)	(M+H4N)+	1	MS/MS	9.6	-14.4	0.315	0.721	0.5	-26.8	0.109	0.49	0.8	-14.5	0.469	0.61	0.6
918.7052	980.8627	13.98	TG 16:0/22:0/22:6	C63H110O6	ESI (+)	(M+H4N)+	1	MS/MS	5.2	-17.6	0.788	0.926	0.8	22.3	0.527	0.72	0.8	48.4	0.031	0.12	1.2
977.8395	978.8467	13.59	TG 18:0/20:1/22:6	C63H108O6	ESI (+)	(M+NH4)+	2	MS/MS	5.6	140.3	0.006	0.010	1.5	87.5	0.006	0.02	1.2	-22.0	0.375	0.53	0.9
868.7506	886.7843	14.19	TG 17:1/18:1/18:2	C56H100O6	ESI (+)	(M+NH4)+	2	MS/MS	5.1	-4.6	1.000	1.000	0.4	20.2	0.315	0.59	0.7	26.1	0.078	0.17	0.9
956.7818	974.8155	13	TG 18:3/20:5/22:1	C63H104O6	ESI (+)	(M+NH4)+	2	MS/MS	7.3	77.1	0.073	0.381	1.1	45.8	0.014	0.04	0.8	-17.7	0.578	0.68	0.7
924.8122	942.8459	13.58	TG 18:1/18:3/21:0	C60H108O6	ESI (+)	(M+NH4)+	3	MS	3.1	0.7	0.788	0.926	0.2	21.5	0.412	0.64	0.6	20.7	0.078	0.17	0.8
774.6758	792.7095	12.68	TG 14:1/16:0/16:1	C49H90O6	ESI (+)	(M+H4N)+	0	MS/MS	6.5	2.0	0.527	0.804	0.3	-21.5	0.412	0.64	0.8	-23.1	0.375	0.53	0.9
947.7938	948.801	13.98	TG 18:1/18:1/22:6	C61H102O6	ESI (+)	(M+H4N)+	1	MS/MS	7.8	41.8	0.527	0.804	0.5	-19.0	0.788	0.88	0.7	-42.9	0.078	0.17	1.1
925.8043	926.8115	13.72	TG 18:1/18:1/20:3	C59H104O6	ESI (+)	(M+H4N)+	2	MS/MS	5.0	-79.8	0.000	0.010	2.2	-83.9	0.000	0.02	2.2	-28.3	0.078	0.17	0.8

Supporting Table S5. Lipids found to be statistically significant for any of the comparisons performed against the healthy control group (AGA).

Mass	*m/z*	RT (min)	Candidate	Formula	Mode	Adduct	Error	Source	CV in QC(%)	SGA vs AGA	p value	pBH	VIP	SGA-GH0m vs AGA	p value	pBH	VIP	SGA-GH3m vs AGA	p value	pBH	VIP
446.3392	445.332	2.42	(24R)-1α,24-dihydroxy-26,27-dimethyl-22-oxavitamin D3 / (24R)-1α,24-dihydroxy-26,27-dimethyl-22-oxacholecalciferol	C28H46O4	ESI (-)	(M-H)-	1	MS/MS	3.5	3.7	1.000	1.000	0.3	-38.8	0.164	0.212	0.6	-18.9	0.788	0.823	0.3
567.422	568.4292	5.41	1-(2-methoxy-tricosanyl)-sn-glycero-3-phosphoethanolamine	C29H62NO7P	ESI (+)	(M+H)+	8	MS/MS	6.0	-90.5	0.016	0.028	1.5	-90.1	0.006	0.012	1.4	-88.7	0.006	0.013	1.4
468.381	467.3738	1.84	17-oxo-hexacosenoic acid	C26H48O3	ESI (-)	(M+C2H3O2)-	1	MS/MS	10.5	-88.0	0.016	0.028	1.3	-84.6	0.006	0.012	1.2	-86.6	0.006	0.013	1.3
362.2429	361.2357	2.62	1a,1b-dihomo-PGJ2 / 1a,1b-dihomo-15-deoxy-delta-12,14-PGD2	C22H34O4	ESI (-)	(M-H)-	8	MS	2.9	-42.0	0.016	0.028	0.7	-57.3	0.006	0.012	0.8	-29.0	0.024	0.040	0.5
340.2406	339.2334	3.21	1-O-(2R-hydroxy-tetradecyl)-sn-glycerol	C17H36O4	ESI (-)	(M+Cl)-	9	MS/MS	0.8	57.6	0.286	0.344	0.9	74.0	0.164	0.212	0.9	33.8	0.230	0.285	0.8
869.7568	870.764	13.58	1-O-eicosanoyl-Cer(d18:1/16:0)	C54H105NO4	ESI (+)	(M+K)+	4	MS	7.6	-14.4	0.730	0.777	0.4	-15.6	0.788	0.812	0.3	19.8	0.527	0.572	0.4
315.314	316.3212	2.98	6-Hydroxy-4-nonadecanone	C19H38O2	ESI (+)	(M+NH4)+	1	MS	2.6	3731.2	0.016	0.028	1.8	5243.9	0.006	0.012	2.0	2476.0	0.042	0.064	1.7
313.2249	314.2321	0.91	ACar 10:1	C17H31NO4	ESI (+)	(M+H)+	0	MS/MS	4.7	-45.0	0.063	0.090	0.6	-48.6	0.042	0.068	0.6	-29.6	0.315	0.376	0.4
367.2729	368.2801	1.16	ACar 14:2	C21H37NO4	ESI (+)	(M+H)+	1	MS/MS	3.4	-57.4	0.016	0.028	0.9	-71.3	0.006	0.012	1.0	-46.6	0.073	0.097	0.6
273.2662	274.2734	1.72	C16 Sphinganine	C16H35NO2	ESI (+)	(M+H)+	2	MS/MS	5.6	-32.2	0.016	0.028	0.8	19.9	0.164	0.212	0.3	-76.2	0.016	0.031	1.1
712.633	711.6258	12.52	CE 18:0	C45H80O2	ESI (-)	(M+C2H3O2)-	6	MS/MS	3.5	-6.3	0.190	0.240	0.4	-14.3	0.230	0.277	0.4	-9.1	0.527	0.572	0.2
693.6616	692.6544	12.54	Cer 18:0;O/24:1	C42H83NO2	ESI (-)	(M+C2H3O2)-	3	MS/MS	21.1	-57.6	0.016	0.028	0.7	-63.9	0.006	0.012	0.9	-52.4	0.006	0.013	0.8
683.6414	682.6342	12.41	Cer 18:0;O2/22:0	C40H81NO3	ESI (-)	(M+C2H3O2)-	1	MS/MS	17.4	-92.5	0.016	0.028	1.3	-92.6	0.006	0.012	1.5	-91.6	0.006	0.013	1.5
653.5959	652.5887	11.93	Cer 18:1;O2/20:0	C38H75NO3	ESI (-)	(M+C2H3O2)-	0	MS/MS	2.8	-53.0	0.016	0.028	0.5	-54.1	0.006	0.012	0.8	-51.9	0.006	0.013	0.8
621.6043	620.5971	12.21	Cer 18:1;O2/22:0	C40H79NO3	ESI (-)	(M-H)-	1	MS/MS	1.4	-15.8	0.905	0.922	0.0	-25.5	0.042	0.068	0.4	-17.5	0.315	0.376	0.3
649.6366	648.6294	12.51	Cer 18:1;O2/24:0	C42H83NO3	ESI (-)	(M-H)-	1	MS/MS	1.3	-17.5	0.413	0.470	0.2	-22.7	0.164	0.212	0.4	-18.5	0.230	0.285	0.3
647.6209	646.6137	12.23	Cer 18:1;O2/24:1	C42H81NO3	ESI (-)	(M-H)-	1	MS/MS	1.3	-23.0	0.905	0.922	0.1	-29.1	0.073	0.107	0.5	-26.8	0.073	0.097	0.4
761.6891	762.6963	12.23	Cer 20:0;O3/26:0;O	C46H93NO5	ESI (+)	(M+Na)+	2	MS/MS	2.2	-92.2	0.016	0.028	1.4	-94.5	0.006	0.012	1.5	-91.4	0.006	0.013	1.4
525.512	526.5192	11.85	Cer 18:0;O2/15:0	C33H67NO3	ESI (+)	(M+H)+	0	MS/MS	0.5	101.5	0.016	0.028	0.8	91.8	0.073	0.107	0.3	81.1	0.073	0.097	0.6
1493.1287	1516.1318	8.09	CL(74:0)	C83H162O17P2	ESI (+)	(M+Na)+	10	MS	1.9	35.6	0.730	0.777	0.4	7.9	0.648	0.686	0.4	62.9	0.073	0.097	0.6
680.5586	679.5514	11.94	DG 16:0/20:2/0:0	C39H72O5	ESI (-)	(M+C2H3O2)-	1	MS/MS	2.0	60.8	0.286	0.344	0.2	40.0	0.164	0.212	0.4	7.6	0.527	0.572	0.1
676.5298	675.5226	10.52	DG 18:1/18:3/0:0	C39H68O5	ESI (-)	(M+C2H3O2)-	3	MS/MS	5.9	129.9	0.032	0.050	0.8	21.5	0.164	0.212	0.5	38.1	0.024	0.040	0.5
200.1775	199.1703	1.46	FA 12:0 Lauric acid	C12H24O2	ESI (-)	(M-H)-	0	MS/MS	4.2	-58.6	0.556	0.611	0.3	-65.8	0.109	0.152	0.6	-50.0	0.788	0.823	0.3
228.2086	227.2014	2.12	FA 14:0 Myristic acid	C14H28O2	ESI (-)	(M-H)-	1	MS/MS	1.5	-35.2	0.063	0.090	0.6	-44.6	0.109	0.152	0.6	-17.9	0.412	0.469	0.3
226.1931	225.1859	1.62	FA 14:1 Myristoleic acid	C14H26O2	ESI (-)	(M-H)-	0	MS/MS	4.6	-34.8	0.032	0.050	0.6	-26.5	0.527	0.576	0.3	-13.5	0.927	0.939	0.3

242.2242	241.217	2.56	FA 15:0	C15H30O2	ESI (-)	(M-H)-	1	MS/MS	17.6	-62.0	0.016	0.028	1.0	-64.3	0.006	0.012	0.9	-56.0	0.006	0.013	0.8
256.2406	255.2334	3.02	FA 16:0 Palmitic acid	C16H32O2	ESI (-)	(M-H)-	2	MS/MS	1.4	-29.0	0.063	0.090	0.4	-44.2	0.006	0.012	0.7	-40.1	0.006	0.013	0.6
254.2243	253.2171	2.33	FA 16:1 Palmitoleic acid	C16H30O2	ESI (-)	(M-H)-	1	MS/MS	1.3	-33.1	0.190	0.240	0.4	-28.0	0.073	0.107	0.4	-8.7	0.412	0.469	0.3
278.2241	277.2169	2.12	FA 18:3 alpha-Linolenic acid	C18H30O2	ESI (-)	(M-H)-	1	MS/MS	1.0	9.3	0.190	0.240	0.3	-20.4	0.164	0.212	0.3	-1.2	0.927	0.939	0.1
313.2981	314.3053	4.07	FA 19:1	C19H36O2	ESI (+)	(M+NH4)+	1	MS/MS	1.8	245.7	0.016	0.028	0.9	305.6	0.006	0.012	1.1	350.8	0.006	0.013	1.1
304.24	303.2328	2.54	FA 20:4 Arachidonic acid	C20H32O2	ESI (-)	(M-H)-	1	MS/MS	2.2	-37.8	0.016	0.028	0.7	-31.1	0.024	0.041	0.5	-25.6	0.109	0.141	0.4
332.2718	331.2646	3.27	FA 22:4 Adrenic acid	C22H36O2	ESI (-)	(M-H)-	1	MS/MS	2.8	23.3	0.190	0.240	0.4	0.8	0.527	0.576	0.1	48.6	0.006	0.013	0.4
330.2558	329.2486	2.76	FA 22:5 DPA	C22H34O2	ESI (-)	(M-H)-	0	MS/MS	1.2	-20.5	0.063	0.090	0.5	-28.6	0.109	0.152	0.4	-13.6	0.315	0.376	0.3
354.3493	353.3421	5.69	FA 23:0	C23H46O2	ESI (-)	(M-H)-	1	MS	7.8	-31.7	1.000	1.000	0.3	-30.8	0.412	0.475	0.5	-38.8	0.042	0.064	0.5
440.3862	439.379	5.08	FA 25:1	C25H48O2	ESI (-)	(M+C2H3O2)-	1	MS/MS	5.2	-47.5	0.016	0.028	0.7	-29.5	0.024	0.041	0.5	-63.8	0.006	0.013	0.9
396.396	395.3888	7.94	FA 26:0	C26H52O2	ESI (-)	(M-H)-	2	MS/MS	8.6	-26.2	0.286	0.344	0.3	-25.9	0.527	0.576	0.4	-20.7	0.527	0.572	0.3
412.3908	411.3836	4.16	FA 26:0;O	C26H52O3	ESI (-)	(M-H)-	2	MS	2.4	-21.1	0.413	0.470	0.5	-6.1	0.230	0.277	0.2	-25.4	0.927	0.939	0.3
280.2408	279.2336	2.62	FA 18:1;O	C18H34O3	ESI (-)	(M-H-H2O)-	4	MS/MS	0.6	-14.8	0.413	0.470	0.2	-32.2	0.073	0.107	0.5	21.0	0.412	0.469	0.3
665.6269	666.6341	12.12	FAHFA(22:1-(18-O-20:0))	C42H80O4	ESI (+)	(M+H4N)+	8	MS/MS	3.7	-46.7	0.016	0.028	1.5	-61.4	0.006	0.012	0.8	-51.0	0.006	0.013	0.7
449.3141	448.3069	0.9	Glycoursodeoxycholic acid	C26H43NO5	ESI (-)	(M-H)-	0	MS/MS	3.0	34.3	0.190	0.240	0.5	-19.9	1.000	1.000	0.2	-60.5	0.024	0.040	0.8
783.6609	784.6681	12	HexCer 18:1/22:0	C46H89NO8	ESI (+)	(M+H)+	5	MS/MS	3.9	-97.6	0.016	0.028	1.3	-94.8	0.006	0.012	1.6	-94.6	0.006	0.013	1.6
837.7089	855.7426	13.96	HexCer 18:1/26:1	C50H95NO8	ESI (+)	(M+NH4)+	4	MS/MS	5.7	-44.5	0.063	0.090	0.6	-52.3	0.012	0.023	0.7	-29.3	0.042	0.064	0.4
763.6287	762.6215	12.51	HexCer 18:2;O2/22:0	C46H87NO8	ESI (-)	(M-H-H2O)-	4	MS/MS	3.2	-68.5	0.016	0.028	0.9	-71.4	0.006	0.012	1.0	-69.8	0.006	0.013	1.0
873.6555	872.6483	11.54	Hex2Cer 18:0;O2/18:0	C48H93NO13	ESI (-)	(M+C2H3O2)-	2	MS	13.3	-88.0	0.016	0.028	1.2	-88.9	0.006	0.012	1.4	-88.3	0.006	0.013	1.4
161.105	162.1122	0.78	L-carnitine	C7H16NO3	ESI (+)	(M+H)+	2	MS/MS	1.7	441.7	0.016	0.028	1.2	421.5	0.006	0.012	1.2	410.4	0.006	0.013	1.2
541.3182	542.3254	1.75	LPC 0:0/20:5	C28H48NO7P	ESI (+)	(M+H)+	1	MS/MS	7.5	-33.9	0.016	0.028	0.9	-43.6	0.073	0.107	0.7	-53.3	0.024	0.040	0.8
481.3164	482.3236	2.18	LPC 15:0/0:0	C23H48NO7P	ESI (+)	(M+H)+	0	MS/MS	4.9	-66.2	0.016	0.028	1.0	-72.4	0.006	0.012	1.0	-61.4	0.012	0.024	0.8
509.348	510.3552	3.04	LPC 17:0/0:0	C25H52NO7P	ESI (+)	(M+H)+	0	MS/MS	3.0	-69.9	0.016	0.028	1.1	-73.7	0.006	0.012	1.0	-64.5	0.012	0.024	0.9
519.3327	520.3399	2.23	LPC 18:2/0:0	C26H50NO7P	ESI (+)	(M+H)+	1	MS/MS	10.8	-12.5	0.032	0.050	0.6	-45.4	0.024	0.041	0.6	-27.6	0.230	0.285	0.5
517.3163	518.3235	1.81	LPC 18:3/0:0	C26H48NO7P	ESI (+)	(M+H)+	1	MS/MS	8.2	-61.9	0.016	0.028	1.0	-71.1	0.006	0.012	1.0	-60.9	0.012	0.024	0.9
567.3329	568.3401	2.09	LPC 22:6/0:0	C30H50NO7P	ESI (+)	(M+H)+	0	MS/MS	15.5	13.6	0.190	0.240	0.4	-35.8	0.230	0.277	0.6	-1.7	1.000	1.000	0.3
270.2555	269.2483	3.49	Methyl hexadecanoate	C17H34O2	ESI (-)	(M-H)-	1	MS	11.7	-54.1	0.016	0.028	0.8	-67.7	0.006	0.012	0.9	-36.1	0.042	0.064	0.5
390.2981	389.2909	3.39	MG 16:0/0:0/0:0	C19H38O4	ESI (-)	(M+C2H3O2)-	0	MS/MS	3.6	51.3	0.032	0.050	0.6	101.2	0.073	0.107	0.7	245.8	0.006	0.013	1.0
418.3292	417.322	4.31	MG 18:0/0:0/0:0	C21H42O4	ESI (-)	(M+C2H3O2)-	0	MS/MS	2.7	1133.1	0.016	0.028	1.5	1388.3	0.073	0.107	1.2	1021.5	0.006	0.013	1.5
788.5216	787.5144	2.56	OxPG 16:0_20:3+1O	C42H77O11P	ESI (-)	(M-H)-	2	MS/MS	6.5	1034.2	0.016	0.028	1.3	979.4	0.006	0.012	1.4	1341.8	0.006	0.013	1.5
764.5231	763.5159	2.6	OxPG 16:0_16:1+1O	C40H77N2O7P	ESI (-)	(M-H)-	0	MS/MS	5.8	420111.1	0.016	0.028	2.6	289046.0	0.006	0.012	2.6	470064.7	0.006	0.013	2.7
745.558	746.5652	9.81	PA 22:2/16:0	C41H77O8P	ESI (+)	(M+NH4)+	6	MS/MS	7.9	-85.4	0.016	0.028	1.4	-81.4	0.006	0.012	1.2	-83.7	0.006	0.013	1.3

805.5632	806.5704	7.55	PC 16:0/22:6	C46H80NO8P	ESI (+)	(M+H)+	1	MS/MS	2.1	112.1	0.032	0.050	0.9	93.4	0.042	0.068	0.8	157.6	0.024	0.040	0.9
865.5826	864.5754	7.34	PC 16:0_22:6	C46H80NO8P	ESI (-)	(M+C2H3O2)-	0	MS/MS	2.2	112.0	0.016	0.028	0.9	72.1	0.024	0.041	0.7	150.4	0.024	0.040	0.9
779.5464	780.5536	6.9	PC 16:0/20:5	C44H78NO8P	ESI (+)	(M+H)+	0	MS/MS	2.9	69.2	0.905	0.922	0.4	38.3	0.230	0.277	0.4	77.6	0.073	0.097	0.7
815.566	814.5588	6.61	PC 16:1/18:2	C44H82NO10P	ESI (-)	(M-H)-	2	MS/MS	5.6	7.4	0.032	0.050	0.5	-4.2	1.000	1.000	0.3	70.8	0.012	0.024	0.7
783.5784	784.5856	8.34	PC 18:1/18:2	C44H82NO8P	ESI (+)	(M+H)+	1	MS/MS	2.9	35.1	0.190	0.240	0.5	24.0	0.109	0.152	0.5	61.8	0.042	0.064	0.6
781.561	782.5682	7.12	PC 18:2/18:2	C44H80NO8P	ESI (+)	(M+H)+	1	MS/MS	1.3	18.3	0.730	0.777	0.4	-5.8	0.527	0.576	0.3	57.5	0.164	0.211	0.6
779.5445	780.5517	6.6	PC 18:2/18:3	C44H78NO8P	ESI (+)	(M+H)+	1	MS/MS	2.0	13.3	0.413	0.470	0.5	3.7	0.042	0.068	0.5	41.4	0.012	0.024	0.6
771.5781	772.5853	9.11	PC 17:0_18:2	C43H82NO8P	ESI (+)	(M+H)+	0	MS/MS	2.6	-1.0	0.286	0.344	0.2	-6.0	0.412	0.475	0.2	39.7	0.024	0.040	0.5
819.5793	820.5865	8.44	PC 17:1_22:5	C47H82NO8P	ESI (+)	(M+H)+	0	MS/MS	3.9	-14.3	0.556	0.611	0.4	9.3	0.527	0.576	0.2	37.6	0.042	0.064	0.5
805.5622	806.5694	6.93	PC 18:2/20:4	C46H80NO8P	ESI (+)	(M+H)+	0	MS/MS	2.7	11.0	0.190	0.240	0.5	-7.3	0.412	0.475	0.5	32.3	0.073	0.097	0.6
831.5779	832.5851	7.77	PC 40:7	C48H82NO8P	ESI (+)	(M+H)+	1	MS/MS	3.7	10.2	0.413	0.470	0.5	1.9	0.412	0.475	0.4	22.5	0.412	0.469	0.6
819.5791	820.5863	8.14	PC 17:0/22:6	C47H82NO8P	ESI (+)	(M+H)+	0	MS/MS	5.2	-31.5	0.063	0.090	0.7	-16.1	0.927	0.950	0.3	0.7	0.788	0.823	0.3
795.5777	796.5849	8.53	PC 17:0/20:4	C45H82NO8P	ESI (+)	(M+H)+	1	MS/MS	3.5	-24.3	0.111	0.155	0.7	-21.7	0.788	0.812	0.5	-19.8	0.648	0.695	0.3
779.5451	780.5523	6.27	PC 18:1_18:4	C44H78NO8P	ESI (+)	(M+H)+	1	MS/MS	3.0	-41.6	0.016	0.028	0.8	-66.7	0.006	0.012	0.9	-33.0	0.230	0.285	0.5
743.5458	744.553	7.25	PC 15:0/18:2	C41H78NO8P	ESI (+)	(M+H)+	0	MS/MS	2.6	-50.4	0.016	0.028	0.8	-54.8	0.006	0.012	0.8	-34.6	0.024	0.040	0.5
719.5462	720.5534	8.22	PC 16:0/15:0	C39H78NO8P	ESI (+)	(M+H)+	0	MS/MS	1.1	-42.2	0.016	0.028	0.8	-37.7	0.042	0.068	0.5	-36.0	0.073	0.097	0.5
771.5784	772.5856	8.77	PC 17:1_18:1	C43H82NO8P	ESI (+)	(M+H)+	0	MS/MS	3.3	-44.3	0.063	0.090	0.8	-39.2	0.527	0.576	0.5	-40.2	0.527	0.572	0.5
773.5945	774.6017	10.47	PC 17:0/18:1	C43H84NO8P	ESI (+)	(M+H)+	0	MS/MS	2.7	-44.7	0.016	0.028	0.9	-35.7	0.648	0.686	0.4	-40.7	0.315	0.376	0.5
729.5306	730.5378	6.54	PC 14:0/18:2	C40H76NO8P	ESI (+)	(M+H)+	0	MS/MS	2.7	-53.2	0.032	0.050	0.7	-60.1	0.012	0.023	0.8	-41.4	0.042	0.064	0.6
769.5609	770.5681	7.72	PC 35:3	C43H80NO8P	ESI (+)	(M+H)+	2	MS/MS	16.2	-58.3	0.016	0.028	0.9	-54.0	0.006	0.012	0.8	-44.1	0.024	0.040	0.7
783.5768	784.584	9.25	PC 16:0/20:3	C44H82NO8P	ESI (+)	(M+H)+	1	MS/MS	3.2	-32.7	0.032	0.050	0.9	-34.1	0.230	0.277	0.5	-48.5	0.073	0.097	0.6
831.5779	832.5851	7.35	PC 18:2/22:5	C48H82NO8P	ESI (+)	(M+H)+	1	MS/MS	4.7	-52.4	0.016	0.028	0.7	-63.1	0.006	0.012	0.9	-51.2	0.024	0.040	0.8
767.5444	768.5516	7.07	PC 15:0/20:4	C43H78NO8P	ESI (+)	(M+H)+	2	MS/MS	6.8	-60.2	0.016	0.028	1.0	-61.7	0.006	0.012	0.9	-53.7	0.006	0.013	0.8
729.5725	730.5797	8.32	PC 15:0/16:0	C39H78NO8P	ESI (+)	(M+H)+	0	MS/MS	5.6	-74.1	0.016	0.028	1.1	-67.1	0.006	0.012	0.9	-53.9	0.006	0.013	0.8
797.5947	798.6019	9.4	PC 19:1/18:2	C45H84NO8P	ESI (+)	(M+H)+	1	MS/MS	2.9	-56.2	0.016	0.028	0.9	-57.7	0.006	0.012	0.8	-54.8	0.006	0.013	0.8
703.5119	704.5191	6.22	PC 16:0/14:1	C38H74NO8P	ESI (+)	(M+H)+	5	MS/MS	8.4	-86.4	0.016	0.028	1.3	-59.3	0.006	0.012	0.8	-59.4	0.012	0.024	0.8
719.5466	720.5538	7.9	PC 18:0/13:0	C39H78NO8P	ESI (+)	(M+H)+	0	MS/MS	1.7	-65.7	0.016	0.028	0.9	-54.9	0.171	0.221	0.6	-60.9	0.073	0.097	0.7
791.5514	792.5586	6.79	PC 37:6	C45H78NO8P	ESI (+)	(M+H)+	3	MS/MS	2.3	-77.3	0.016	0.028	1.2	-80.4	0.006	0.012	1.2	-65.7	0.006	0.013	1.0
777.5395	778.5467	6.17	PC 36:6	C44H76NO8P	ESI (+)	(M+H)+	7	MS/MS	5.7	-78.7	0.016	0.028	1.0	-83.3	0.006	0.012	1.2	-71.7	0.006	0.013	1.1
799.611	800.6182	11.18	PC 19:0/18:2	C45H86NO8P	ESI (+)	(M+H)+	0	MS/MS	7.1	-88.6	0.016	0.028	0.9	-83.8	0.006	0.012	1.3	-74.4	0.006	0.013	1.1
831.5767	832.5839	7.96	PC 20:4/20:3	C48H82NO8P	ESI (+)	(M+H)+	1	MS/MS	19.4	-83.4	0.016	0.028	1.2	-85.7	0.006	0.012	1.3	-75.8	0.006	0.013	1.1
807.5781	808.5853	8.34	PC 38:5	C46H82NO8P	ESI (+)	(M+H)+	2	MS/MS	4.1	-85.1	0.016	0.028	1.2	-85.8	0.006	0.012	1.3	-81.6	0.006	0.013	1.2

779.5462	780.5534	7.38	PC 16:1_20:4	C44H78NO8P	ESI (+)	(M+H)+	0	MS/MS	18.4	-95.5	0.016	0.028	1.6	-92.8	0.006	0.012	1.5	-94.3	0.006	0.013	1.6
765.555	766.5622	7.87	PC 18:3/P-18:1	C44H80NO7P	ESI (+)	(M+H)+	1	MS/MS	1.4	-96.6	0.016	0.028	1.7	-91.5	0.006	0.012	1.3	-97.6	0.006	0.013	1.7
757.5623	758.5695	7.86	PC 16:1/18:1	C42H80NO8P	ESI (+)	(M+H)+	0	MS/MS	2.9	-99.5	0.016	0.028	2.1	-99.6	0.006	0.012	2.1	-99.4	0.006	0.013	2.1
715.5462	716.5534	8.52	PC O-14:0/18:2	C40H78NO7P	ESI (+)	(M+H)+	8	MS/MS	26.9	-65.8	0.016	0.028	1.0	-54.9	0.006	0.012	0.8	-58.5	0.006	0.013	0.9
741.5683	742.5755	9.07	PC O-16:1/18:2	C42H80NO7P	ESI (+)	(M+H)+	1	MS/MS	3.2	-22.0	0.730	0.777	0.4	-33.7	0.230	0.277	0.6	1.1	0.527	0.572	0.3
793.598	794.6052	10.56	PC O-18:0/20:5	C46H84NO7P	ESI (+)	(M+H)+	1	MS/MS	4.0	-71.7	0.016	0.028	1.0	-75.6	0.006	0.012	1.1	-68.7	0.006	0.013	1.0
819.6137	820.6209	9.84	PC O-18:0/22:6	C48H86NO7P	ESI (+)	(M+H)+	1	MS/MS	7.9	-69.0	0.016	0.028	1.0	-47.9	0.164	0.212	0.6	-55.2	0.073	0.097	0.7
797.6306	798.6378	11.58	PC P-16:0/22:2	C46H88NO7P	ESI (+)	(M+H)+	1	MS/MS	7.5	-92.5	0.556	0.611	0.3	-89.7	0.006	0.012	1.4	-89.8	0.006	0.013	1.4
755.5898	756.597	10.28	PC P-18:0/17:2	C43H82NO7P	ESI (+)	(M+H)+	9	MS/MS	7.6	-90.7	0.016	0.028	1.4	-92.8	0.006	0.012	1.5	-89.2	0.006	0.013	1.4
763.5204	764.5276	7.82	PE 16:0/22:6	C43H74NO8P	ESI (+)	(M+H)+	0	MS/MS	20.7	-99.9	0.016	0.028	2.2	-98.7	0.006	0.012	1.9	-99.7	0.006	0.013	2.2
745.5606	746.5678	8.52	PE 18:0_18:1	C41H80NO8P	ESI (+)	(M+H)+	2	MS/MS	3.1	-47.9	0.063	0.090	0.7	-46.0	0.024	0.041	0.6	-37.0	0.109	0.141	0.6
767.5462	768.5534	10.47	PE 18:0_20:4	C43H74NO8P	ESI (+)	(M+H)+	1	MS/MS	4.1	665.3	0.016	0.028	1.3	590.7	0.006	0.012	1.2	632.1	0.006	0.013	1.3
767.5456	766.5384	10.09	PE 18:1/20:3	C43H78NO8P	ESI (-)	(M-H)-	1	MS/MS	3.3	66.3	0.016	0.028	0.8	70.0	0.006	0.012	0.6	61.3	0.006	0.013	0.6
753.5667	752.5595	11.46	PE O-18:0/20:4	C43H80NO7P	ESI (-)	(M-H)-	1	MS/MS	24.9	-57.3	0.032	0.050	0.7	-59.3	0.006	0.012	0.8	-17.5	0.042	0.064	0.3
747.5192	746.512	8.47	PE P-16:0/22:6	C43H74NO7P	ESI (-)	(M-H)-	1	MS/MS	3.0	149.9	0.016	0.028	1.0	69.7	0.024	0.041	0.7	158.9	0.006	0.013	0.9
727.5501	726.5429	11.28	PE P-18:0/18:2	C41H78NO7P	ESI (-)	(M-H)-	2	MS/MS	1.9	-85.4	0.016	0.028	1.2	-88.1	0.006	0.012	1.3	-91.9	0.006	0.013	1.5
751.5509	750.5437	11.28	PE P-18:0/20:4	C43H78NO7P	ESI (-)	(M-H)-	0	MS/MS	1.6	72.7	0.032	0.050	0.8	61.3	0.042	0.068	0.6	67.8	0.024	0.040	0.6
749.5355	750.5427	9.1	PE P-18:0/20:5	C43H76NO7P	ESI (+)	(M+H)+	1	MS/MS	3.4	-98.5	0.016	0.028	1.8	-99.0	0.006	0.012	2.0	-97.0	0.006	0.013	1.7
760.5302	778.5639	11.52	PG 20:1/15:1	C41H77O10P	ESI (+)	(M+H4N)+	6	MS/MS	6.7	-95.7	0.016	0.028	1.6	-93.2	0.006	0.012	1.5	-93.7	0.006	0.013	1.6
862.5569	861.5497	7.24	PI 18:2/18:0	C45H83O13P	ESI (-)	(M-H)-	0	MS/MS	1.6	-17.3	0.190	0.240	0.5	-16.9	0.648	0.686	0.3	-15.8	0.230	0.285	0.3
775.4387	774.4315	12.01	PS 18:4/18:4	C42H66NO10P	ESI (-)	(M-H)-	5	MS/MS	8.5	-28.9	0.016	0.028	0.3	12.8	1.000	1.000	0.1	55.9	0.024	0.040	0.5
839.5661	838.5589	6.73	PS 40:4	C46H82NO10P	ESI (-)	(M-H)-	2	MS	3.5	106.6	0.286	0.344	0.5	105.2	0.006	0.012	0.8	117.0	0.012	0.024	0.8
702.567	703.5742	7.09	SM 18:1;O2/16:0	C39H79N2O6P	ESI (+)	(M+H)+	1	MS/MS	3.0	-99.6	0.016	0.028	0.5	-99.6	0.006	0.012	2.2	-99.5	0.006	0.013	2.2
858.6803	857.6731	11.73	SM 17:1;O2/24:1	C46H91N2O6P	ESI (-)	(M+C2H3O2)-	1	MS/MS	4.5	-84.2	0.016	0.028	1.3	-83.9	0.006	0.012	1.2	-84.7	0.006	0.013	1.3
840.7088	841.716	12.25	SM 18:1;O2/26:1	C49H97N2O6P	ESI (+)	(M+H)+	0	MS/MS	11.7	-68.3	0.016	0.028	0.9	-75.4	0.006	0.012	1.1	-76.0	0.006	0.013	1.1
844.6654	843.6582	11.38	SM 18:2;O2/22:0	C45H89N2O6P	ESI (-)	(M+C2H3O2)-	2	MS/MS	4.1	-84.6	0.016	0.028	1.3	-86.0	0.006	0.012	1.3	-85.1	0.006	0.013	1.3
716.5819	717.5891	7.86	SM 18:1;O2/17:0	C40H81N2O6P	ESI (+)	(M+H)+	1	MS/MS	2.6	-57.5	0.016	0.028	1.1	-51.7	0.012	0.023	0.7	-50.5	0.024	0.040	0.7
786.6529	787.6601	11.63	SM 18:1;O2/22:0	C45H91N2O6P	ESI (+)	(M+H)+	2	MS/MS	2.9	-96.8	0.016	0.028	1.6	-97.9	0.006	0.012	1.8	-96.8	0.006	0.013	1.7
874.7136	873.7064	12.19	SM 18:1;O2/24:0	C47H95N2O6P	ESI (-)	(M+C2H3O2)-	1	MS/MS	1.3	-12.1	0.905	0.922	0.3	-20.9	0.230	0.277	0.4	-19.1	0.412	0.469	0.3
378.3141	377.3069	4.31	ST 24:0;O3	C24H42O3	ESI (-)	(M-H)-	2	MS	5.2	152.8	0.016	0.028	0.9	244.0	0.006	0.012	1.0	146.6	0.006	0.013	0.8
949.9006	950.9078	14.03	TG 18:0/18:0/21:0	C60H116O6	ESI (+)	(M+NH4)+	3	MS/MS	12.7	-98.5	0.016	0.028	1.6	-98.3	0.006	0.012	1.9	-98.1	0.006	0.013	1.9
871.7619	872.7691	14.23	TG 16:0/18:2/18:2	C55H98O6	ESI (+)	(M+NH4)+	1	MS/MS	8.7	-97.8	0.016	0.028	1.4	-97.3	0.006	0.012	1.7	-97.5	0.006	0.013	1.8

842.6768	843.684	14.49	TG 48:1	C51H96O6	ESI (+)	(M+K)+	0	MS	7.0	-98.7	0.016	0.028	1.9	-97.2	0.006	0.012	1.8	-98.5	0.006	0.013	1.9
828.7172	829.7244	15.06	TG 48:0	C51H98O6	ESI (+)	(M+H4N)+	1	MS/MS	10.3	-97.3	0.016	0.028	1.7	-97.0	0.006	0.012	1.7	-97.0	0.006	0.013	1.8
981.8709	982.8781	14.84	TG 18:0/20:0/22:5	C63H112O6	ESI (+)	(M+H4N)+	2	MS/MS	5.2	-96.8	0.016	0.028	1.6	-95.4	0.006	0.012	1.6	-96.6	0.006	0.013	1.7
930.7654	931.7726	14.83	TG 56:5	C59H104O6	ESI (+)	(M+Na)+	1	MS/MS	3.4	-91.5	0.016	0.028	1.2	-93.1	0.006	0.012	1.4	-92.8	0.006	0.013	1.4
964.6995	965.7067	13.26	TG 58:10	C61H98O6	ESI (+)	(M+K)+	8	MS	7.8	-91.1	0.016	0.028	1.5	-91.7	0.006	0.012	1.5	-86.3	0.006	0.013	1.3
900.7174	901.7246	13.58	TG 16:0/18:2/20:4	C57H98O6	ESI (+)	(M+Na)+	0	MS/MS	2.8	-89.4	0.016	0.028	1.4	-90.4	0.006	0.012	1.4	-84.3	0.012	0.024	1.3
925.8054	881.7002	13.51	TG 18:0/16:1/17:2	C54H98O6	ESI (+)	(M+K)+	1	MS/MS	6.6	-90.6	0.016	0.028	1.3	-89.7	0.006	0.012	1.4	-87.7	0.006	0.013	1.3
898.7035	899.7107	13.3	TG 54:7	C57H96O6	ESI (+)	(M+Na)+	1	MS	4.2	-87.6	0.016	0.028	1.2	-89.6	0.006	0.012	1.3	-72.0	0.024	0.040	0.9
890.6711	891.6783	13.58	TG 18:2/16:1/18:2	C55H96O6	ESI (+)	(M+K)+	7	MS/MS	3.4	-87.8	0.016	0.028	1.3	-89.5	0.006	0.012	1.3	-84.0	0.006	0.013	1.3
927.8257	928.8329	15.56	TG 18:1/18:1/20:2	C59H106O6	ESI (+)	(M+H4N)+	1	MS/MS	19.1	-93.2	0.016	0.028	1.1	-89.3	0.006	0.012	1.4	-84.0	0.006	0.013	1.2
977.843	978.8502	13.98	TG 18:4/20:1/22:2	C63H108O6	ESI (+)	(M+NH4)+	2	MS/MS	9.1	-94.8	0.016	0.028	1.5	-87.4	0.006	0.012	1.3	-94.8	0.006	0.013	1.6
865.8081	866.8153	16.26	TG 16:0/17:0/18:0	C54H104O6	ESI (+)	(M+H4N)+	2	MS/MS	25.2	-90.4	0.016	0.028	1.3	-82.8	0.006	0.012	1.2	-79.4	0.006	0.013	1.2
834.7676	857.7529	15.82	TG 13:0/16:0/21:0	C53H102O6	ESI (+)	(M+Na)+	5	MS/MS	24.6	3.7	0.413	0.5	0.8	-80.7	0.109	0.2	0.5	-84.3	0.412	0.5	0.6
767.6995	768.7067	13.75	TG 14:0/14:0/16:0	C47H90O6	ESI (+)	(M+H4N)+	1	MS/MS	7.9	-88.4	0.016	0.028	1.3	-80.0	0.006	0.012	1.2	-84.2	0.006	0.013	1.3
916.6918	917.699	13.78	TG 16:1/18:2/20:3	C57H98O6	ESI (+)	(M+K)+	1	MS/MS	4.3	-82.9	0.016	0.028	1.1	-74.2	0.006	0.012	1.0	-79.1	0.006	0.013	1.1
939.8169	940.8241	13.73	TG 17:0/18:0/22:5	C60H106O6	ESI (+)	(M+NH4)+	9	MS/MS	12.0	-74.7	0.016	0.028	1.0	-73.5	0.006	0.012	1.0	-67.5	0.006	0.013	0.9
979.8555	913.797	13.98	TG 14:1/22:0/o-18:0	C57H110O5	ESI (+)	(M+K)+	2	MS/MS	18.5	-59.6	0.016	0.028	0.7	-69.6	0.006	0.012	1.0	-72.1	0.006	0.013	1.0
837.7779	838.7851	15.26	TG 16:0/16:0/17:0	C52H100O6	ESI (+)	(M+H4N)+	1	MS/MS	26.3	-63.6	0.016	0.028	1.0	-66.4	0.006	0.012	0.9	-61.2	0.006	0.013	0.9
927.8248	928.832	15.2	TG 18:1/18:1/20:2	C59H106O6	ESI (+)	(M+H4N)+	1	MS/MS	10.7	-76.2	0.413	0.470	0.4	-63.4	0.073	0.107	0.9	-61.7	0.073	0.097	0.9
845.7463	846.7535	13.1	TG 14:0/18:1/18:2	C53H96O6	ESI (+)	(M+H4N)+	0	MS/MS	22.5	-72.2	0.016	0.028	1.0	-59.3	0.006	0.012	0.8	-58.6	0.006	0.013	0.9
817.7158	818.723	13.55	TG 48:3	C51H92O6	ESI (+)	(M+H4N)+	1	MS/MS	13.1	-62.6	0.016	0.028	0.8	-53.2	0.012	0.023	0.7	-64.8	0.006	0.013	0.9
923.7856	924.7928	13.08	TG 18:0/18:1/20:5	C59H102O6	ESI (+)	(M+NH4)+	10	MS/MS	10.8	-50.0	0.016	0.028	0.7	-50.2	0.006	0.012	0.7	-39.7	0.006	0.013	0.7
925.807	926.8142	14.54	TG 18:1/18:1/20:3	C59H104O6	ESI (+)	(M+H4N)+	2	MS/MS	9.3	-60.2	0.063	0.090	0.8	-47.9	0.527	0.576	0.5	-17.8	1.000	1.000	0.7
843.7309	844.7381	13.51	TG 14:0/18:2/18:2	C53H94O6	ESI (+)	(M+H4N)+	1	MS/MS	10.4	-54.4	0.016	0.028	0.7	-47.8	0.024	0.041	0.7	-33.7	0.042	0.064	0.5
927.8251	928.8323	13.91	TG 18:0/18:3/20:1	C59H106O6	ESI (+)	(M+NH4)+	1	MS/MS	5.4	-47.2	0.016	0.028	0.6	-47.0	0.012	0.023	0.7	-34.1	0.024	0.040	0.5
929.8403	930.8475	15.74	TG 18:1/18:1/20:1	C59H108O6	ESI (+)	(M+H4N)+	1	MS/MS	10.3	-43.3	0.063	0.090	0.3	-46.7	0.006	0.012	0.7	-49.0	0.006	0.013	0.7
1033.9046	1034.9118	14.65	TG 20:0/22:1/22:6	C67H116O6	ESI (+)	(M+H4N)+	1	MS/MS	17.3	-19.9	0.905	0.922	0.4	-44.6	0.012	0.023	0.7	-46.6	0.006	0.013	0.7
899.7944	900.8016	14.45	TG 18:1/18:1/18:2	C57H102O6	ESI (+)	(M+H4N)+	1	MS/MS	7.5	3.3	0.556	0.611	0.5	-35.5	0.164	0.212	0.6	-24.0	0.412	0.469	0.3
871.7635	872.7707	13.96	TG 16:1/18:1/18:2	C55H98O6	ESI (+)	(M+H4N)+	1	MS/MS	8.0	-29.0	0.556	0.611	0.4	-34.1	0.230	0.277	0.5	-0.8	0.927	0.939	0.4
912.7898	952.8277	13.96	TG 18:0/20:1/20:5	C61H106O6	ESI (+)	(M+NH4)+	5	MS/MS	5.6	-1.3	1.000	1.000	0.4	-21.7	1.000	1.000	0.3	29.5	0.109	0.141	0.5
901.8102	902.8174	15.03	TG 18:1/18:1/18:1	C57H104O6	ESI (+)	(M+H4N)+	1	MS/MS	7.5	-16.3	0.063	0.090	0.5	-21.6	0.527	0.576	0.4	-14.3	0.527	0.572	0.3
925.8081	926.8153	14.66	TG 16:0/18:1/22:4	C59H104O6	ESI (+)	(M+H4N)+	1	MS/MS	8.6	-14.9	0.905	0.922	0.5	-10.8	0.315	0.376	0.4	17.3	0.042	0.064	0.5

936.8146	954.8482	13.96	TG 58:5	C61H108O6	ESI (+)	(M+NH4)+	0	MS	5.6	5.3	0.190	0.240	0.5	-2.3	0.230	0.277	0.4	40.3	0.006	0.013	0.6
897.7786	898.7858	13.98	TG 18:1/18:2/18:2	C57H100O6	ESI (+)	(M+H4N)+	1	MS/MS	8.1	10.6	0.905	0.922	0.5	1.1	0.788	0.812	0.4	58.8	0.109	0.141	0.6
925.8091	926.8163	14.83	TG 18:0/18:1/20:4	C59H104O6	ESI (+)	(M+H4N)+	1	MS/MS	9.6	-14.1	0.190	0.240	0.5	1.5	0.648	0.686	0.5	-13.3	0.315	0.376	0.4
918.7052	980.8627	13.98	TG 16:0/22:0/22:6	C63H110O6	ESI (+)	(M+H4N)+	1	MS/MS	5.2	14.1	0.730	0.777	0.5	2.4	0.648	0.686	0.4	52.0	0.073	0.097	0.6
977.8395	978.8467	13.59	TG 18:0/20:1/22:6	C63H108O6	ESI (+)	(M+NH4)+	2	MS/MS	5.6	-3.4	0.190	0.240	0.7	13.5	0.073	0.107	0.5	-11.4	0.648	0.695	0.5
868.7506	886.7843	14.19	TG 17:1/18:1/18:2	C56H100O6	ESI (+)	(M+NH4)+	2	MS/MS	5.1	21.8	0.413	0.470	0.3	27.3	0.024	0.041	0.4	60.5	0.006	0.013	0.6
956.7818	974.8155	13	TG 18:3/20:5/22:1	C63H104O6	ESI (+)	(M+NH4)+	2	MS/MS	7.3	40.3	0.286	0.344	0.3	44.1	0.109	0.152	0.4	18.6	0.927	0.939	0.3
924.8122	942.8459	13.58	TG 18:1/18:3/21:0	C60H108O6	ESI (+)	(M+NH4)+	3	MS	3.1	57.9	0.016	0.028	0.6	65.3	0.006	0.012	0.6	99.5	0.006	0.013	0.7
774.6758	792.7095	12.68	TG 14:1/16:0/16:1	C49H90O6	ESI (+)	(M+H4N)+	0	MS/MS	6.5	74.0	0.190	0.240	0.5	69.5	0.073	0.107	0.4	30.4	0.788	0.823	0.0
947.7938	948.801	13.98	TG 18:1/18:1/22:6	C61H102O6	ESI (+)	(M+H4N)+	1	MS/MS	7.8	55.0	0.111	0.155	0.9	160.5	0.012	0.023	0.9	48.8	0.006	0.013	0.8
925.8043	926.8115	13.72	TG 18:1/18:1/20:3	C59H104O6	ESI (+)	(M+H4N)+	2	MS/MS	5.0	2303479.5	0.016	0.028	3.0	3375636.4	0.006	0.012	2.9	2691507.4	0.006	0.013	2.9
